# Prevalence of low-grade inflammation in depression: a systematic review and meta-analysis of CRP levels

**DOI:** 10.1017/S0033291719001454

**Published:** 2019-07-01

**Authors:** Emanuele Felice Osimo, Luke James Baxter, Glyn Lewis, Peter B. Jones, Golam M. Khandaker

**Affiliations:** 1 Department of Psychiatry, School of Clinical Medicine, University of Cambridge, Cambridge, UK; 2 Cambridgeshire and Peterborough NHS Foundation Trust, Cambridge, UK; 3 MRC London Institute of Medical Sciences, Faculty of Medicine, Imperial College London, Hammersmith Hospital Campus, London, UK; 4 School of Clinical Medicine, University of Cambridge, Cambridge, UK; 5 Division of Psychiatry, University College London, London, UK

**Keywords:** C-reactive protein, CRP, depression, immunopsychiatry, inflammation, low-grade inflammation, meta-analysis, mood, prevalence, review

## Abstract

**Background:**

Peripheral low-grade inflammation in depression is increasingly seen as a therapeutic target. We aimed to establish the prevalence of low-grade inflammation in depression, using different C-reactive protein (CRP) levels, through a systematic literature review and meta-analysis.

**Methods:**

We searched the PubMed database from its inception to July 2018, and selected studies that assessed depression using a validated tool/scale, and allowed the calculation of the proportion of patients with low-grade inflammation (CRP >3 mg/L) or elevated CRP (>1 mg/L).

**Results:**

After quality assessment, 37 studies comprising 13 541 depressed patients and 155 728 controls were included. Based on the meta-analysis of 30 studies, the prevalence of low-grade inflammation (CRP >3 mg/L) in depression was 27% (95% CI 21–34%); this prevalence was not associated with sample source (inpatient, outpatient or population-based), antidepressant treatment, participant age, BMI or ethnicity. Based on the meta-analysis of 17 studies of depression and matched healthy controls, the odds ratio for low-grade inflammation in depression was 1.46 (95% CI 1.22–1.75). The prevalence of elevated CRP (>1 mg/L) in depression was 58% (95% CI 47–69%), and the meta-analytic odds ratio for elevated CRP in depression compared with controls was 1.47 (95% CI 1.18–1.82).

**Conclusions:**

About a quarter of patients with depression show evidence of low-grade inflammation, and over half of patients show mildly elevated CRP levels. There are significant differences in the prevalence of low-grade inflammation between patients and matched healthy controls. These findings suggest that inflammation could be relevant to a large number of patients with depression.

## Introduction

Depression is a common mental illness with a complex aetiology and is one of the leading causes of disability worldwide, affecting around 10–20% of the general population in their lifetime (Lim *et al*., 2018). There is now increasing evidence suggesting an association between depression and inflammation (Goldsmith *et al*., 2016). For instance, ‘sickness behaviour’ commonly seen following an acute infection, shares many characteristics with depression, such as fatigue, sleep disturbance and decreased motivation (Dantzer *et al*., 2008); early-life infection and autoimmune diseases are associated with a higher risk of depression in adulthood (Benros *et al*., 2013); people with chronic immune-mediated inflammatory diseases such as rheumatoid arthritis exhibit a higher prevalence of depression (Dickens *et al*., 2002). Depression is also associated with other conditions linked with elevated inflammatory markers, such as cardiovascular disease (CVD) (Ridker, 2003).

C-reactive protein (CRP) is a marker of acute phase response which has been used most extensively as a measure of low-grade inflammation in psychiatric (von Känel *et al*., 2007; Fernandes *et al*., 2016) and physical conditions (Visser *et al*., 1999; Danesh *et al*., 2000). CRP is associated with cardiovascular risk, including myocardial infarction, stroke, sudden cardiovascular death and peripheral vascular disease (Ridker, 2003). Meta-analyses of cross-sectional studies confirm that mean concentrations of circulating CRP and inflammatory cytokines such as interleukin 6 (IL-6) are higher in patients with acute depression compared with controls (Howren *et al*., 2009; Dowlati *et al*., 2010; Haapakoski *et al*., 2015; Goldsmith *et al*., 2016). Population-based longitudinal studies show that higher levels of CRP and IL-6 at baseline are associated with an increased risk of depression in subsequent follow-ups (Gimeno *et al*., 2009; Wium-Andersen *et al*., 2013; Khandaker *et al*., 2014; Zalli *et al*., 2016), suggesting that inflammation could be a cause rather than simply a consequence of the illness.

The association between inflammation and depression is clinically relevant. Poor response to antidepressants is associated with the activation of inflammatory immune responses (Lanquillon *et al*., 2000; Benedetti *et al*., 2002; Carvalho *et al*., 2013; Chamberlain *et al*., 2018). It has been reported the mean CRP levels are higher in treatment-resistant compared with treatment-responsive patients with depression (Maes *et al*., 1997; Sluzewska *et al*., 1997). Anti-inflammatory treatment has antidepressant effects (Müller *et al*., 2006; Köhler *et al*., 2014; Kappelmann *et al*., 2018). Randomised controlled trials (RCTs) indicate that anti-inflammatory drugs are likely to be beneficial particularly for depressed patients who show evidence of inflammation (Raison *et al*., 2013; Kappelmann *et al*., 2018). Currently, a number of ongoing RCTs of anti-inflammatory treatments are recruiting specifically depressed patients with elevated CRP levels (e.g. ⩾3 mg/L): NCT02473289; ISRCTN16942542 (Khandaker *et al*., 2018). Therefore, a better understanding of the prevalence of low-grade inflammation in depression, and of factors associated with inflammation could inform future research and clinical practice.

Inflammation is unlikely to be relevant for all patients with depression (Khandaker *et al*., 2017). While it is established that mean concentrations of peripheral inflammatory markers are higher in depressed patients compared with controls (Howren *et al*., 2009; Dowlati *et al*., 2010; Haapakoski *et al*., 2015; Goldsmith *et al*., 2016), it is unclear what *proportion* of depressed patients show evidence of low-grade inflammation. Many studies have reported on the prevalence of inflammation in depressed patients using various CRP level thresholds to define inflammation, e.g. >3 or >1 mg/L. These studies have been conducted in different settings and populations, e.g. inpatient, outpatient, population-based (Raison *et al*., 2013; Wium-Andersen *et al*., 2013; Shin *et al*., 2016). The reported prevalence of inflammation varies widely among these studies; for example, for low-grade inflammation (CRP >3 mg/L) it has been reported to vary between 0% and 60% in existing studies (Ma *et al*., 2011; Hannestad *et al*., 2013). However, as far as we are aware, a systematic review and meta-analysis of the prevalence of low-grade inflammation in patients with depression is currently lacking. While it is likely that the prevalence of low-grade inflammation is higher in patients with depression compared with controls, to our knowledge, no systematic review and meta-analysis has examined the odds ratio for inflammation in depressed patients compared with matched controls.

We conducted a systematic review of existing studies to: (1) quantify the prevalence of low-grade inflammation in patients with depression using meta-analysis; (2) calculate the odds ratio for low-grade inflammation in depressed patients compared with matched healthy controls using meta-analysis; (3) identify sociodemographic and other factors associated with inflammation prevalence in patients with depression using meta-regression analysis. We defined low-grade inflammation as serum CRP levels >3 mg/L. This cut-off has been chosen based on the American Heart Association and Center for Disease Control and Prevention recommendations, which defined CRP levels of >3 mg/L as high (Pearson *et al*., 2003). In addition, we carried out additional analyses using CRP levels >1 mg/L to define ‘elevated CRP’, and >10 mg/L to define ‘very high CRP’ indicative of current infection. We also carried out a number of sensitivity analyses; for example, meta-analyses using >1 and >3 mg/L thresholds for CRP were repeated using only studies that excluded patients with suspected infection (defined as CRP >10 mg/L); and after excluding poor quality studies.

## Methods

### Search strategy and study selection

This systematic review has been performed according to the Preferred Reporting Items for Systematic Reviews and Meta-analyses (PRISMA) guidelines. The search protocol was prospectively published on PROSPERO (see: http://www.crd.york.ac.uk/PROSPERO/display_record.asp?ID=CRD42018106640). The PubMed database was searched for published studies from its inception to 5 of July 2018 using the following keywords: ‘(*CRP OR* “*C-reactive protein*” *OR* “*hs-CRP*” *OR hsCRP*) *OR* (*C-Reactive Protein*[*mesh*] AND depressi*)’. No language restriction was applied; we only selected studies based on human participants. The electronic search was complemented by hand-searching of meta-analyses and review articles. Abstracts were screened, and full texts of relevant studies were retrieved. Two authors applied the inclusion/exclusion criteria independently and selected the final studies for this review (LB and EFO).

### Selection criteria

We included studies that: (1) examined CRP levels in people with depression; (2) assessed depression using clinical criteria (DSM or ICD) or a validated tool (e.g. Hamilton Depression Rating Scale), and reported it as a categorical variable (yes/no); (3) reported CRP levels allowing the calculation of the proportion of ‘inflamed’ patients using cut-offs of either 3, 1 or 10 mg/L. One study used CRP cut-offs of 0.99 and 3.13 mg/L, which was included (Penninx *et al*., 2003), as these values are very close to the thresholds above. Exclusion criteria were (1) studies reporting measures of inflammation other than CRP, e.g. interleukins or genetic markers; (2) *in vitro* or animal studies; (3) non-original data, e.g. reviews; (4) studies exclusively based on patients with a medical condition, e.g. cancer.

### Recorded variables

The main outcome measure was the proportion of subjects showing elevated CRP in patients and, where reported, in non-depressed controls. We also extracted the following data: author; year of publication; sampling criteria; diagnostic criteria for depression; age of participants; treatment status (antidepressant-free, treatment resistant); ethnicity; matching criteria for patients and controls (if present); study setting and sample source (e.g. community or inpatient); presence of comorbidities. If there were multiple publications from the same data set, we used the study with the largest sample.

### Data synthesis

We performed meta-analyses of the prevalence of inflammation in depressed patients using three different CRP cut-offs to define inflammation: >3 (primary), >1 and >10 mg/L. The pooled prevalence of inflammation was calculated using quantitative random-effect meta-analysis, expressed as percentage and 95% CI. The use of random-effect meta-analysis, as opposed to fixed effect, is appropriate when there is heterogeneity between studies. Pooling of studies was performed using the inverse variance method, so that studies with bigger samples were given greater weight. The Clopper–Pearson method was used to compute confidence interval for individual studies, and the logit transformation was used for the transformations of proportions, with a continuity correction of 0.5 in studies with zero cell frequencies. Heterogeneity between studies was measured using the *I*^2^ statistic, which describes the percentage of the variability in effect estimates that is due to heterogeneity. Heterogeneity was tested using Cochrane's *Q*-Test (Higgins and Thompson, 2002). Publication bias was assessed for each group of studies by visual inspection of funnel plots, and tested with an Egger's regression test for funnel plot asymmetry (mixed-effects meta-regression model). *P* values <0.05, two tailed, were considered statistically significant. We used meta-regression analyses to evaluate the association of inflammation prevalence with age, sex, body mass index (BMI), sample source, proportion of antidepressant-free patients and ethnicity. Seventeen studies reported CRP levels in matched non-depressed controls; these were used to calculate the meta-analytic odds ratio for inflammation in patients with depression *v*. healthy controls using random-effects estimates for meta-analyses with binary outcome data; pooling of studies was performed using the inverse variance method and with a continuity correction of 0.5 in studies with zero cell frequencies. Study quality was assessed using the Newcastle–Ottawa Scale (Stang, 2010). Analyses were repeated with poor quality studies removed. Meta-analyses were carried out using the *meta* package [version 4.9 (Schwarzer, 2007)] in R 3.4 (R Core Team, 2017), and plotted using packages *meta* and *Cairo* v1.5 (Urbanek and Horner, 2015). Additional information on the methods can be found in the Supplementary Materials.

## Results

The literature search yielded 1545 results, out of which 37 studies met the inclusion criteria for meta-analysis (Legros *et al*., 1985; Penninx *et al*., 2003; Ladwig *et al*., 2005; Liukkonen *et al*., 2006; O'brien *et al*., 2006; Almeida *et al*., 2007; Kling *et al*., 2007; Danese *et al*., 2008; Nilsson *et al*., 2008; Cizza *et al*., 2009; Harley *et al*., 2010; Ma *et al*., 2011; Naghashpour *et al*., 2011; Hannestad *et al*., 2013; Raison *et al*., 2013; Shanahan *et al*., 2013; Park *et al*., 2014; Uher *et al*., 2014; Wium-Andersen *et al*., 2014; Courtet *et al*., 2015; Wysokiński *et al*., 2015; Cepeda *et al*., 2016; Haroon *et al*., 2016; Rapaport *et al*., 2016; Shin *et al*., 2016; Ekinci and Ekinci, 2017; Euteneuer *et al*., 2017; Gallagher *et al*., 2017; Horsdal *et al*., 2017; Jha *et al*., 2017; Cáceda *et al*., 2018; Chamberlain *et al*., 2018; Felger *et al*., 2018; Osimo *et al*., 2018*b*; Porcu *et al*., 2018; Shibata *et al*., 2018; Wei *et al*., 2018). Please see Supplementary Fig. S1 for the PRISMA diagram of study selection, and Table 1 for details of the included studies.

**Table 1.** Characteristics of studies included in the meta-analysis

Study	Country	Setting	Depressed (*N*)	Controls (*N*)	Mean age of patients in years (SD)	Patient sex (% male)	Assessment of depression^a^	Quality rating^b^
Legros *et al*. (1985)	Belgium	Inpatient	34	NA^c^	42 (NA)	NA	Feighner *et al*. (1972) criteria(Feighner *et al*., 1972)	Good
Penninx *et al*. (2003)	USA	Prospective /population-based	145	2879	74 (2.9)	38.62	CES-D	Good
Ladwig *et al*. (2005)	Germany	Prospective /population-based	986	2035	57.5 (7.8)	100	Subscale from the von Zerssen affective symptom checklist(von Zerssen and Cording, 1978); depression = score ⩾11	Good
Liukkonen *et al*. (2006)	Finland	Prospective /population-based	962	4097	31 (0)	39.4	Hopkins symptom checklist-25 (Parloff *et al*., 1954)	Good
O'Brien *et al*. (2006)	Ireland	Outpatient	32	20	44.05 (NA)	34.38	DSM-IV	Poor
Almeida *et al*. (2007)	Australia	Prospective /population-based	213	4000	76.6 (4.4)	100	GDS-15 score ⩾7	Good
Kling *et al*. (2007)	USA and Israel	Outpatient	18	18	41 (12)	0	DSM-IV	Good
Danese *et al*. (2008)	UK	Prospective /population-based	109	673	32 (0)	39.45	DSM-IV	Good
Nilsson *et al*. (2008)	Sweden	Outpatient	50	NA	Median age 71 years	NA	DSM IV	Good
Cizza *et al*. (2009)	USA	Outpatient	77	41	35.5 (7)	0	DSM-IV SCI	Good
Harley *et al*. (2010)	New Zealand	Outpatient	346	NA	NA	NA	DSM-IV SCI, Montgomery Asberg Depression Rating Scale	Poor
Ma *et al*. (2011)	USA	Outpatient	10	498	NA	30	BDI score ⩾22	Poor
Naghashpour *et al*. (2011)	Iran	Outpatient	43	52	37.26 (6.5)	0	BDI score >5	Poor
Hannestad *et al*. (2013)	USA	Outpatient	9	7	37 (14.3)	44.44	DSM-IV	Fair
Raison *et al*. (2013)	USA	Outpatient	60	NA	43.4 (8.8)	33.33	DSM-IV depression through SCID	Good
Shanahan *et al*. (2013)	USA	Prospective /population-based	61	NA	13.5 (1.9)	NA	DSM-IV criteria assessed through CAPA	Good
Park *et al*. (2014)	Korea	Outpatient	30	30	65.2 (4.8)	30	DSM-IV SCI	Good
Uher *et al*. (2014)	Canada, UK, Germany, Croatia, Denmark, Slovenia, Belgium	Outpatient	241	NA	40.7 (11.4)	37.34	Schedule for Clinical Assessment in Neuropsychiatry	Good
Wium-Andersen *et al*. (2014)	Denmark	Prospective /population-based	1183	77 626	65 (NA)	36.35	ICD criteria	Poor
Wysokiński *et al*. (2015)	Poland	Inpatient	319	NA	59.7 (21)	23.82	ICD-10	Poor
Courtet *et al*. (2015)	France	Inpatient	600	NA	39.8 (13.4)	27.83	DSM-IV	Good
Cepeda *et al*. (2016)	USA	Prospective /population-based	1325	12 951	45.3 (12)	35.92	PHQ9 score >9	Good
Haroon *et al*. (2016)	USA	Outpatient	50	NA	38.6 (10.8)	32	DSM-IV	Fair
Rapaport *et al*. (2016)	USA	Outpatient	155	NA	46.1(12.6)	41.29	DSM-IV SCID, Clinical global impressions severity score, HAMD-17	Fair
Shin *et al*. (2016)	Korea	Outpatient	2492	49 736	36.75 (6.52)	66.77	CES-D score ⩾21	Poor
Ekinci and Ekinci (2017)	Turkey	Inpatient	139	50	42.2 (12.3)	30.21	DSM-IV	Poor
Euteneuer *et al*. (2017)	Germany	Outpatient	98	30	37.3 (12.2)	51.02	DSM-IV	Good
Gallagher *et al*. (2017)	Canada	Prospective /population-based	811	5084	67.3 (10.8)	32.18	CES-D ⩾4	Poor
Horsdal *et al*. (2017)	Denmark	Prospective /population-based	2187	NA	Median age 35.7 years	37.4	ICD-8 and ICD-10	Good
Jha *et al*. (2017)	USA, Singapore	Outpatient	106	NA	46.64 (11.89)	30.19	International Neuropsychiatric Interview (MINI)	Good
Wei *et al*. (2018)	China	Inpatient	18	15	43.89 (20.78)	27.78	DSM-IV	Good
Cáceda *et al*. (2018)	USA	Inpatient	52	NA	36.8 (12.6)	36.54	DSM-IV	Good
Chamberlain *et al*. (2018)	UK	Outpatient	198	54	36.5 (NA)	15.15	DSM-5	Good
Felger *et al*. (2018)	USA	Outpatient	73	NA	42.1 (11.1)	42.47	SCID-IV	Good
Osimo *et al*. (2018*b*)	UK	Inpatient	137	NA	40(13)	47.44	ICD-10	Good
Porcu *et al*. (2018)	Brazil	Outpatient	67	NA	46 (NA)	NA	DSM-5, ICD-10	Good
Shibata *et al*. (2018)	Japan	Prospective /population-based	105	2424	65.5 (11.1)	NA	CESD score ⩾16	Poor

a CES-D, The Center for Epidemiologic Studies Depression Scale; DSM-IV, Diagnostic and Statistical Manual of Mental Disorders, 4th. Edition; GDS, Geriatric Depression Scale; BDI, Beck's Depression Inventory; SCID, Structured Clinical Interview for DSM; CAPA, The Child and Adolescent Psychiatric Assessment; ICD, World Health Organisation International Classification of Diseases; PHQ9, Patient Health Questionnaire-9; HAMD-17, Hamilton Depression Rating Scale (HDRS).

b Studies evaluated using the Newcastle–Ottawa Scale (see Supplementary methods and Table S1), then converted to Agency for Healthcare Research and Quality – AHRQ – standards (good, fair and poor) using these thresholds:
• Good quality: ⩾75% in Selection domain AND ⩾50% in Comparability domain AND ⩾50% in Outcome domain. • Fair quality: 50% in Selection domain AND ⩾50% in Comparability domain AND ⩾50% in Outcome domain. • Poor quality: ⩽50% in Selection domain OR 0% in Comparability domain OR ⩽50% in Outcome domain.

c Not available.

### Prevalence of low-grade inflammation (CRP >3 mg/L) in depressed patients

#### Results based on all available studies

**Fig. 1.**
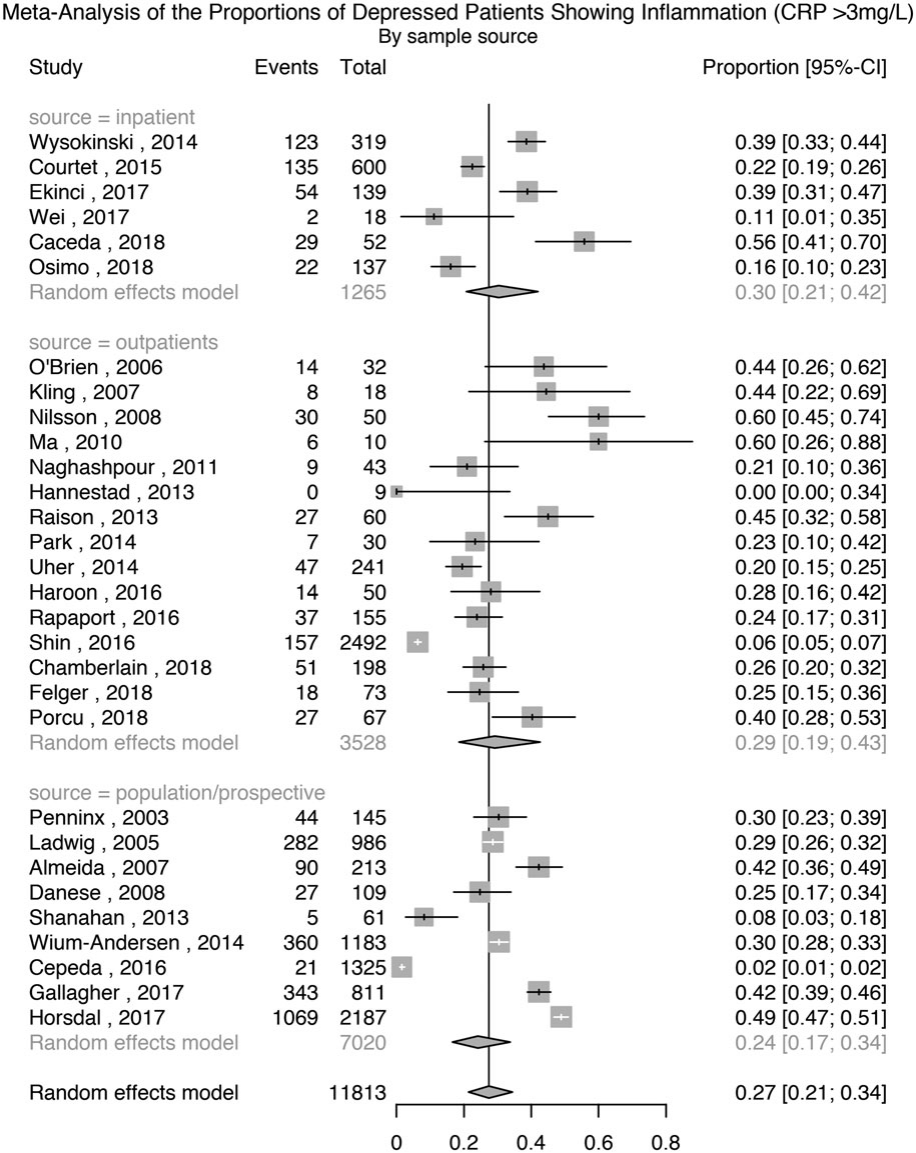
Prevalence of low-grade inflammation (CRP >3 mg/L) in depressed patients.

Thirty studies comprising 11 813 patients with depression were used for this analysis. The meta-analytic pooled prevalence of low-grade inflammation in depressed patients was 27% (95% CI 21–34%); see Fig. 1. There was evidence of heterogeneity among studies (*I*^2^ = 97.7%; 95% CI 97.3–98.1%; Cochrane's *Q* = 1264; *p* = <0.01). Further analyses after grouping studies by setting showed that the prevalence of inflammation in inpatient samples (*N* = 1265) was 30% (95% CI 21–42%; *I*^2^ = 91.9%; Cochrane's *Q* = 62); in outpatient samples (*N* = 3528) it was 29% (95% CI 19–43%; *I*^2^ = 95.9%; Cochrane's *Q* = 338); and in population-based samples (*N* = 7020) it was 24% (95% CI 17–34%; *I*^2^ = 98.3%; Cochrane's *Q* = 483).

#### Analyses excluding poor quality studies or cases with past depression

A sensitivity analysis excluding six poor quality studies, comprising 8778 patients, showed that the prevalence of inflammation was 27% (95% CI 22–33%); Supplementary Table S1 and Fig. S2. There was evidence of heterogeneity (*I*^2^ = 96.4%; 95% CI 95.5–97.1%; Cochrane's *Q* = 644; *p* = <0.01). A sensitivity analysis excluding two studies where depression was not active in all patients, comprising 11 763 patients, showed that the prevalence of inflammation was 26% (95% CI 20–34%); Supplementary Fig. S3. There was evidence of heterogeneity (*I*^2^ = 97.9%; 95% CI 97.4–98.2%; Cochrane's *Q* = 1261; *p* = <0.01).

#### Analysis after excluding cases of suspected infection (CRP >10 mg/L)

Nine studies reported the prevalence of low-grade inflammation after excluding participants with suspected infection, defined as CRP >10 mg/L. A separate meta-analysis based on these studies, comprising 6948 patients, showed that the prevalence of inflammation was 16% (95% CI 8–32%); Supplementary Fig. S4. There was evidence of heterogeneity (*I*^2^ = 98.8%; 95% CI 98.5–99.1%; Cochrane's *Q* = 675; *p* = <0.01).

#### Association between prevalence of low-grade inflammation (CRP >3 mg/L) and characteristics of depressed patients

Meta-regression was used on 19 studies comprising 7858 patients to test the association between the prevalence of inflammation and the proportion of patients who were antidepressant-free at the time of study. There was no association between these factors (estimate: −0.007; *z* = −1.03; *p* = 0.30). Similarly, sex, age, non-White ethnicity, BMI and sample source (inpatient, outpatient or population-based) were not associated with the prevalence of inflammation (see Supplementary Results).

#### Assessment of publication bias

A funnel plot of the 30 studies assessing the prevalence of low-grade inflammation (CRP >3 mg/L) in depression visually appeared symmetrical (Supplementary Fig. S5). Egger's regression test for funnel plot asymmetry was non-significant (*t* = −1.3; df = 28; *p* = 0.21), suggesting there was no evidence of publication bias.

#### Odds ratio for low-grade inflammation (>3 mg/L) in depressed patients

**Fig. 2.**
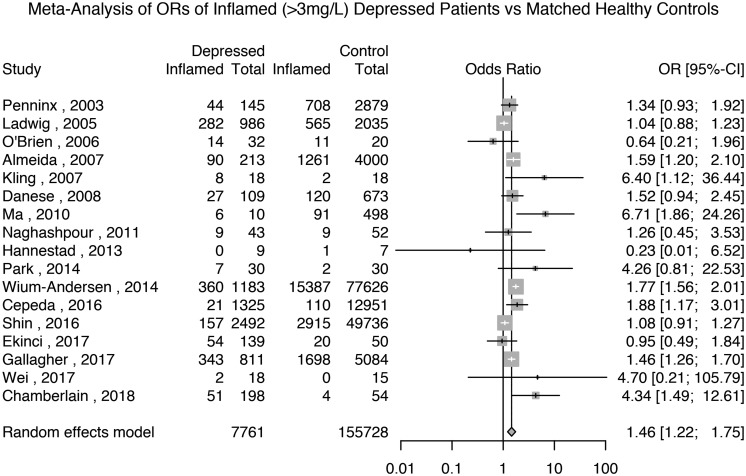
Odds ratio for low-grade inflammation (CRP >3 mg/L) in depressed patients compared with matched controls.

Seventeen studies reported the prevalence of inflammation in 7761 depressed patients and 155 728 matched non-depressed controls (see Supplementary Table S2 for matching details). The meta-analytic OR for inflammation in depressed patients compared with matched controls was 1.46 (95% CI 1.22–1.75; *p* < 0.0001); see Fig. 2. There was evidence of heterogeneity (*I*^2^ = 71.9%; 95% CI 54.3–82.7%; Cochrane's *Q* = 57; *p* = <0.01).

Based on the same studies, we meta-analysed the prevalence of inflammation in depressed patients and matched non-depressed controls separately. The prevalence of inflammation in controls was 16% (95% CI 11–23%) and that in depressed patients was 24% (95% CI 17–34%); see Supplementary Figs S6 and S7.

We carried out sensitivity analyses based on five available studies of depressed patients and matched healthy controls that excluded subjects with very high levels of CRP (>10 mg/L). These studies, comprising 3868 patients and 63 212 controls, showed that the prevalence of inflammation in controls was 10% (95% CI 3–26%) and that in patients it was 13% (95% CI 4–36%); see Supplementary Figs S8 and S9. Based on these studies, the meta-analytic OR for inflammation in depressed patients compared with matched controls was 1.44 (95% CI 0.80–2.61; *p* = 0.23); see Supplementary Fig. S10.

A sensitivity analysis excluding poor quality studies, comprising 5045 patients and 105 372 controls, showed that the meta-analytic OR for inflammation in depressed patients compared with matched controls was 1.56 (95% CI 1.29–1.88; *p* < 0.0001); see Supplementary Table S1 and Fig. S11. A further sensitivity analysis only including studies that matched patients and controls by BMI, comprising 2624 patients and 79 887 controls, showed that the meta-analytic OR for inflammation in depressed patients compared with matched controls was 1.59 (95% CI 1.08–2.34; *p* = 0.02); see Supplementary Fig. S12. Finally, a sensitivity analysis excluding studies where depression was not active in all patients showed that the meta-analytic OR for inflammation in depressed patients compared with matched controls was 1.47 (95% CI 1.22–1.75; *p* = 0.02); see Supplementary Fig. S13.

### Prevalence of elevated CRP levels (>1 mg/L) in depressed patients

#### Results based on all available studies

**Fig. 3.**
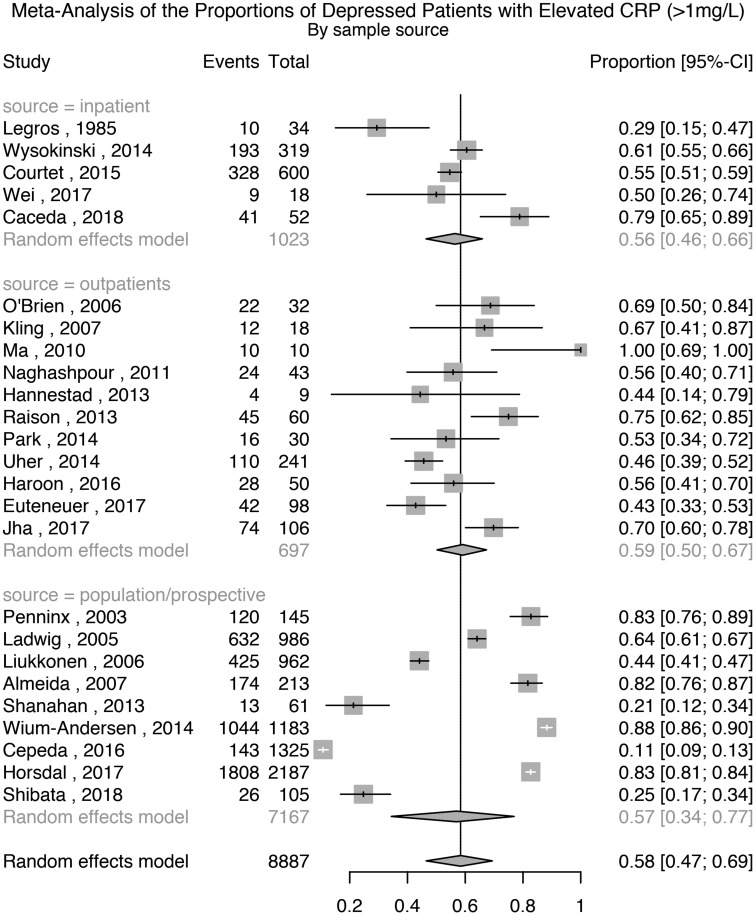
Prevalence of elevated CRP (>1 mg/L) in depressed patients.

Twenty-five studies comprising 8887 patients with depression were used for this analysis. The meta-analytic pooled prevalence of elevated CRP >1 mg/L in depressed patients was 58% (95% CI 47–69%); see Fig. 3. There was evidence of heterogeneity (*I*^2^ = 98.7%; 95% CI 98.5–98.9%; Cochrane's *Q* = 1862; *p* = <0.01). Further analyses after grouping studies by setting showed that the prevalence of elevated CRP in inpatient samples (*N* = 1023) was 56% (95% CI 46–66%; *I*^2^ = 81.8%; Cochrane's *Q* = 22); in outpatient samples (*N* = 697) was 59% (95% CI 50–67%; *I*^2^ = 74.9%; Cochrane's *Q* = 40); and in population-based samples (*N* = 7167) was 57% (95% CI 34–77%; *I*^2^ = 99.5%; Cochrane's *Q* = 1774).

#### Analyses excluding poor quality studies or cases with past depression

A sensitivity analysis excluding four poor quality studies showed that the prevalence of elevated CRP >1 mg/L in depressed patients was 57% (95% CI 43–69%); Supplementary Table S1 and Fig. S14. There was evidence of heterogeneity (*I*^2^ = 98.9%; 95% CI 98.7–99.1%; Cochrane's *Q* = 1858; *p* = <0.01). A further sensitivity analysis excluding studies where depression was not active in all patients showed that the prevalence of elevated CRP in depressed patients was 58% (95% CI 45–69%); Supplementary Fig. S15. There was evidence of heterogeneity (*I*^2^ = 98.8%; 95% CI 98.6–99.0%; Cochrane's *Q* = 1861; *p* = <0.01).

#### Analysis after excluding cases of suspected infection (CRP>10 mg/L)

Eight studies also reported the prevalence of elevated CRP after excluding participants with suspected infection, defined as CRP >10 mg/L. A separate meta-analysis based on these studies, comprising 4456 patients that excluded patients with CRP levels >10 mg/L showed that the prevalence of elevated CRP >1 mg/L was 50% (95% CI 29–72%); see Supplementary Fig. S16 There was evidence of heterogeneity (*I*^2^ = 99.1%; 95% CI 98.9–99.3%; Cochrane's *Q* = 816; *p* = <0.01).

#### Association between prevalence of elevated CRP levels (>1 mg/L) and characteristics of depressed patients

Meta-regression analyses did not find any significant association of elevated CRP with sex, age, BMI, non-White ethnicity, being antidepressant-free or sample source (see Supplementary Results).

#### Assessment of publication bias

A funnel plot of the 25 studies assessing the prevalence of elevated CRP in depression appeared visually symmetrical. Egger's regression test for funnel plot asymmetry was non-significant (*t* = −0.43; df = 23; *p* = 0.67), suggesting there was no evidence of publication bias (Supplementary Fig. S17).

#### Odds ratio for elevated CRP levels (>1 mg/L) in depressed patients

**Fig. 4.**
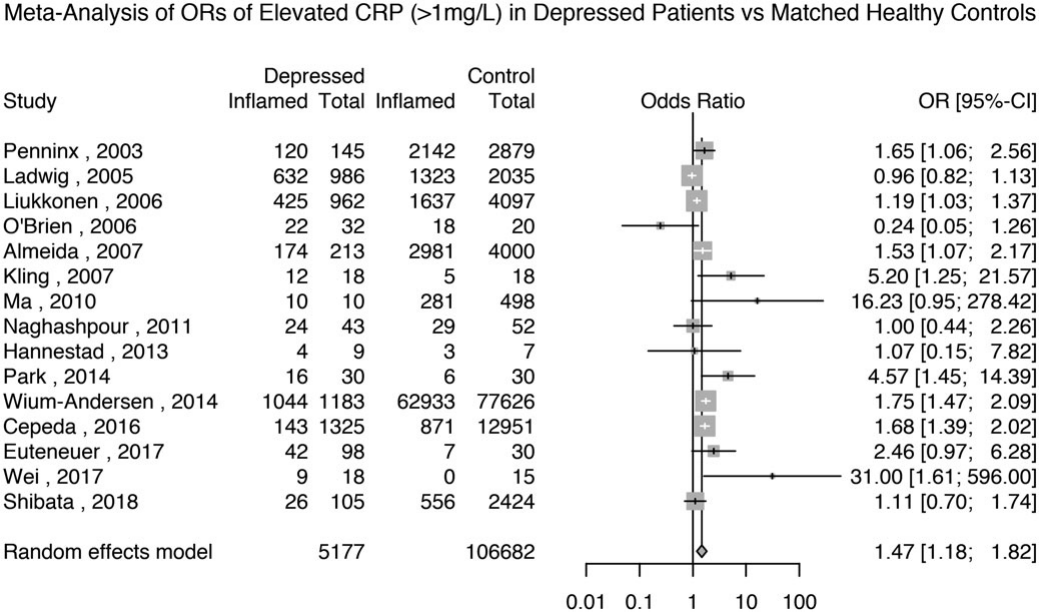
Odds ratio for elevated CRP (>1 mg/L) in depressed patients compared with matched controls.

Fifteen studies reported the prevalence of elevated CRP >1 mg/L in 5177 depressed patients and 106 682 matched non-depressed controls (see Supplementary Table S2 for matching details). The meta-analytic OR for elevated CRP in depressed patients compared with matched controls was 1.47 (95% CI 1.18–1.82; *p* = 0.0005); see Fig. 4. There was evidence of heterogeneity (*I*^2^ = 75.6%; 95% CI 59.8–85.2%; Cochrane's *Q* = 57; *p* = <0.01).

Based on the same studies, we meta-analysed the prevalence of elevated CRP in depressed patients and matched non-depressed controls separately. The prevalence of elevated CRP >1 mg/L in controls was 44% (95% CI 26–65%) and that in depressed patients was 59% (95% CI 41–75%); see Supplementary Figs S18 and S19.

A sensitivity analysis based on 12 studies after excluding poor quality studies showed that the meta-analytic OR for elevated CRP in depressed patients compared with matched controls was 1.51 (95% CI 1.22–1.88; *p* = 0.0002); see Supplementary Table S1 and Fig. S20. A sensitivity analysis only including the nine studies that matched the patients and controls by BMI showed that the meta-analytic OR for elevated CRP >1 mg/L in depressed patients compared with matched controls was 1.52 (95% CI 1.12–2.07; *p* = 0.01); see Supplementary Fig. S21. A further sensitivity analysis excluding studies where depression was not active in all patients showed that the meta-analytic OR for elevated CRP in depressed patients compared with healthy controls was 1.47 (95% CI 1.19–1.81; *p* = 0.0003); see Supplementary Fig. S22. Finally, a sensitivity analysis of the four studies excluding subjects with very high levels of CRP (>10 mg/L) showed that the meta-analytic OR for elevated CRP in depressed patients compared with healthy controls was 1.29 (95% CI 0.38–4.30; *p* = 0.68); see Supplementary Fig. S23.

### Very high CRP levels (>10 mg/L) in depressed patients and healthy controls

We used data from four available studies comprising 3926 patients and 62 748 matched healthy controls. The meta-analytic pooled prevalence of very high CRP in depressed patients matched to healthy controls was 3% (95% CI 1–11%); in the same studies, prevalence of very high CRP in healthy controls matched to depressed patients was 1% (95% CI 0–4%); the meta-analytic OR for very high CRP in depressed patients compared with matched controls was 1.52 (95% CI 1.13–2.05; *p* = 0.006); see Supplementary Figs S24–26.

## Discussion

To our knowledge, this is one of the first systematic reviews and meta-analyses of the prevalence of low-grade inflammation in patients with depression. We report that a notable proportion of depressed patients show evidence of inflammation. Approximately one in four patients with depression show CRP levels >3 mg/L, a widely used threshold to define low-grade inflammation in the literature. The prevalence is unaltered after excluding poor quality studies, or after excluding studies where depression was not active. After excluding patients with suspected infection, the prevalence of low-grade inflammation is about one in six. We also report that approximately three patients out of five have mildly elevated CRP (>1 mg/L). The prevalence is unaltered after excluding poor quality studies, or after excluding studies where depression was not active. After excluding patients with suspected infection, the prevalence of elevated CRP is one in two. Meta-regression analyses show that the prevalence of inflammation in depression is not associated with sex, age, BMI, ethnicity or sample source.

Using matched non-depressed controls, we quantified the odds ratios of low-grade inflammation and of elevated CRP in depressed patients. We report that the proportion of patients with depression showing elevated inflammatory markers as compared to matched healthy controls is remarkably stable: the ORs were 1.46 for CRP levels >3 mg/L, 1.47 for CRP levels >1 mg/L and 1.52 for CRP levels >10 mg/L. There was no evidence of publication bias within the included studies, but there was evidence of heterogeneity in all analyses.

Knowing inflammation levels in patients with depression could be important for several reasons, particularly for predicting the risk of physical illness and for predicting response to psychiatric treatment. Inflammation is a potentially causal risk factor for CVD (Pearson *et al*., 2003), because CVD is associated with circulating IL-6 and CRP levels (Pradhan *et al*., 2002; Danesh *et al*., 2004; Danesh *et al*., 2008) and with genetic variants regulating levels/activity of IL-6 (IL6R Genetics Consortium Emerging Risk Factors Collaboration, 2012; Interleukin-6 Receptor Mendelian Randomisation Analysis Consortium, 2012). Depression is co-morbid with CVD (Hare *et al*., 2013). Depression increases the risk of incident CVD, and is a marker of poor prognosis after myocardial infarction (Nicholson *et al*., 2006). Inflammation could be a shared mechanism for these conditions. Using Mendelian randomisation analysis of the UK Biobank sample, we previously found that out of all cardiovascular risk factors, IL-6, CRP and triglycerides are likely to be causally linked with depression (Khandaker *et al*., 2019). Therefore, cardiovascular risk screening in depressed patients who show evidence of inflammation could be useful. Our work suggests that such screening will be relevant for about a quarter of patients with depression.

We focussed on CRP levels as our preferred measure of inflammation because it has been widely used in different fields of medicine to measure inflammation, and standardised cut-offs for CRP exist in the literature. The American Heart Association and Center for Disease Control and Prevention have proposed clear CRP thresholds as indicators of inflammation levels (<1 = ‘low’, 1–3 = ‘medium’, >3 mg/L = ‘high’) (Pearson *et al*., 2003). Our findings are consistent with previous meta-analyses reporting higher mean concentrations of CRP, IL-6 and other inflammatory markers in depressed patients compared with controls (Howren *et al*., 2009; Dowlati *et al*., 2010; Haapakoski *et al*., 2015; Goldsmith *et al*., 2016). Our study adds to the literature by providing information on the proportion of depressed patients who have evidence of inflammation.

In addition to depression and CVD, inflammation is associated with other physical and psychiatric disorders including diabetes mellitus (Pradhan *et al*., 2001), schizophrenia (Miller *et al*., 2011; Khandaker *et al*., 2015) and dementias (Schmidt *et al*., 2002). Inflammation is also an important predictor of increased all-cause mortality (Zacho *et al*., 2010; Sung *et al*., 2014; Li *et al*., 2017). Therefore, routine CRP screening in patients with depression, and identification and treatment of the cause of inflammation could improve overall health-related mortality and morbidity. Public health interventions aimed at reducing inflammation could improve mortality and morbidity associated with a number of conditions.

It is unlikely that anti-inflammatory drugs will be useful for all patients with depression (Khandaker *et al*., 2017). Measurement of CRP levels could inform patient selection in RCTs of anti-inflammatory drugs for depression. We are aware of two studies that are testing novel anti-inflammatory drugs such as monoclonal antibodies (mAb) against the IL-6/IL-6R pathway. One study testing the efficacy and safety of sirukumab (anti-IL-6 mAb) for depression has completed recruitment (NCT02473289). We are conducting an RCT of tocilizumab (anti-IL-6R mAb) for patients with depression (Khandaker *et al*., 2018). Both of these studies are based on patients with CRP levels ⩾3 mg/L. Secondary analysis of existing RCTs suggests mAb against specific inflammatory cytokines, such as IL-6/IL-6R, could be helpful for depression (Sun *et al*., 2017; Kappelmann *et al*., 2018). However, definitive efficacy trials need to be completed before anti-inflammatory drugs can be considered in psychiatric clinical practice. Our findings suggest that up to a quarter of depressed patients show signs of low-grade inflammation. Future studies should explore the potential causes for this, and also whether depressed patients with higher CRP levels may benefit from anti-inflammatory treatments.

Studies included in this review varied on setting, country and analytic methods, and the proportion of depressed patients with elevated CRP (>3 mg/L) in these studies ranged between 0% and 60%. In our analyses, the prevalence of inflammation was not associated with participant age and sex, antidepressant treatment, ethnicity or source of sample. This is the case despite the samples spanning all age groups [median age: 42.2 years; inter-quartile range (IQR): 37–59]. Both sexes were well represented (median proportion of males: 36%, IQR: 17–41%). The samples comprised both antidepressant-free and treated populations (antidepressant-free = 6 studies; 100% treated = 3 studies; mixed populations = 10 studies). Included studies covered samples collected from inpatient (*N* = 6), outpatient (*N* = 15) and general population (*N* = 9). One reason for not detecting an association between inflammation and sociodemographic factors could be that a number of studies matched patients and controls on these factors.

The meta-analytic prevalence of low-grade inflammation (CRP >3 mg/L) in non-depressed controls seen in our analysis is 16%, which is lower than the prevalence of inflammation reported in some general population studies. For instance, Ford *et al*. (2004) reported the prevalence of low-grade inflammation to be about 25% in a sample of adult US women. Khera *et al*. (2005) reported the prevalence of CRP >3 mg/L to be >30% in US adult males and females. One reason for these high prevalence reports in general population samples could be that these studies include both healthy and diseased individuals including those with chronic inflammatory physical illness. Therefore, for a more accurate comparison of the prevalence of inflammation between depressed cases and healthy controls, we have used studies that included cases matched to healthy controls for the calculation of odds ratios. In our results, the stability of the odds ratios for elevated CRP in depressed patients compared with healthy controls across different CRP thresholds (ORs = 1.46 for CRP levels >3 mg/L; OR = 1.47 for CRP levels >1 mg/L; and OR = 1.52 for CRP levels >10 mg/L) provides confidence that patients are more likely to have evidence of inflammation than healthy controls. Furthermore, excluding patients with very high levels of inflammation did not significantly affect the odds ratio for low-grade inflammation (>3 mg/L) in depressed subjects (OR = 1.44).

Strengths of this work include the systematic nature of the literature search, which identified a large number of relevant studies comprising 13 541 patients and 155 728 controls from different countries and settings, and spanning diverse ethnic and age groups. The methods were laid out prospectively and published on PROSPERO (Osimo *et al*., 2018*a*). We assessed the studies for quality using the validated Newcastle–Ottawa Scale (Stang, 2010). We conducted multiple sensitivity analyses to examine the robustness of the findings. There was no evidence of publication bias, suggesting that we covered a range of results spanning the whole expected distribution of means.

Limitations of this work include sample heterogeneity: the studies we included used different methods to assess depression (albeit a valid method was required for inclusion), and samples were recruited from different sources making it difficult to test the association between the prevalence of inflammation and depression severity. However, we have reported meta-analytic results separately by sample source (community, inpatient, etc.), which could be taken as an indirect indicator of depression severity. Inflammation prevalence did not differ much by sample source. However, due to the lack of comparable data on depression severity, we could not assess this directly. Study setting and sample characteristics could account for some of the observed heterogeneity. We used random-effects meta-analyses in order to take care of inter-study variability. Another limitation is that we were not able to account for comorbidities, partly because for some studies this information was not reported. By design we have focused on dichotomous measure of inflammation, so we cannot comment on the distributions of continuous CRP values in patients/controls; these have been subject to previous meta-analyses reporting higher mean levels of CRP in depression compared with controls (Howren *et al*., 2009; Dowlati *et al*., 2010; Haapakoski *et al*., 2015; Goldsmith *et al*., 2016).

In summary, this systematic review and meta-analysis provides a robust estimate of the prevalence of low-grade inflammation in depressed patients, which is about one in four. We also report that depressed patients are about 50% more likely to have evidence of inflammation as compared to matched non-depressed controls. These findings are relevant for future treatment studies of anti-inflammatory drugs and for clinical practice, particularly for predicting response to antidepressants and for predicting co-morbid, immune-related physical illness, such as CVD.

## Supplementary material

For supplementary material accompanying this paper visit https://doi.org/10.1017/S0033291719001454. click here to view supplementary material

## References

[ref1] Almeida OP, Flicker L, Norman P, Hankey GJ, Vasikaran S, van Bockxmeer FM and Jamrozik K (2007) Association of cardiovascular risk factors and disease with depression in later life. The American Journal of Geriatric Psychiatry 15, 506–513. 17158633 10.1097/01.JGP.0000246869.49892.77

[ref2] Benedetti F, Lucca A, Brambilla F, Colombo C and Smeraldi E (2002) Interleukine-6 serum levels correlate with response to antidepressant sleep deprivation and sleep phase advance. Progress in Neuro-psychopharmacology and Biological Psychiatry 26, 1167–1170. 12452541 10.1016/s0278-5846(02)00255-5

[ref3] Benros ME, Waltoft BL, Nordentoft M, Østergaard SD, Eaton WW, Krogh J and Mortensen PB (2013) Autoimmune diseases and severe infections as risk factors for mood disorders: a nationwide study. JAMA Psychiatry 70, 812–820. 23760347 10.1001/jamapsychiatry.2013.1111

[ref4] Cáceda R, Griffin WST and Delgado PL (2018) A probe in the connection between inflammation, cognition and suicide. Journal of Psychopharmacology 32, 482–488. 29552947 10.1177/0269881118764022 PMC9230995

[ref5] Carvalho L, Torre J, Papadopoulos A, Poon L, Juruena M, Markopoulou K, Cleare A and Pariante C (2013) Lack of clinical therapeutic benefit of antidepressants is associated overall activation of the inflammatory system. Journal of Affective Disorders 148, 136–140. 23200297 10.1016/j.jad.2012.10.036

[ref6] Cepeda MS, Stang P and Makadia R (2016) Depression is associated with high levels of C-reactive protein and low levels of fractional exhaled nitric oxide: results from the 2007–2012 national health and nutrition examination surveys. The Journal of Clinical Psychiatry 77, 1666–1671. 27337107 10.4088/JCP.15m10267

[ref7] Chamberlain SR, Cavanagh J, de Boer P, Mondelli V, Jones DN, Drevets WC, Cowen PJ, Harrison NA, Pointon L and Pariante CM (2018) Treatment-resistant depression and peripheral C-reactive protein. The British Journal of Psychiatry, 1–9. 10.1192/bjp.2018.66 PMC6124647 29764522

[ref8] Cizza G, Eskandari F, Coyle M, Krishnamurthy P, Wright E, Mistry S and Csako G (2009) Plasma CRP levels in premenopausal women with major depression: a 12-month controlled study. Hormone and Metabolic Research 41, 641. 19408214 10.1055/s-0029-1220717 PMC2782561

[ref9] Courtet P, Jaussent I, Genty C, Dupuy A, Guillaume S, Ducasse D and Olie E (2015) Increased CRP levels may be a trait marker of suicidal attempt. European Neuropsychopharmacology 25, 1824–1831. 26032768 10.1016/j.euroneuro.2015.05.003

[ref10] Danese A, Moffitt TE, Pariante CM, Ambler A, Poulton R and Caspi A (2008) Elevated inflammation levels in depressed adults with a history of childhood maltreatment. Archives of General Psychiatry 65, 409–415. 18391129 10.1001/archpsyc.65.4.409 PMC2923056

[ref11] Danesh J, Whincup P, Walker M, Lennon L, Thomson A, Appleby P, Gallimore JR and Pepys MB (2000) Low grade inflammation and coronary heart disease: prospective study and updated meta-analyses. British Medical Journal 321, 199–204. 10903648 10.1136/bmj.321.7255.199 PMC27435

[ref12] Danesh J, Wheeler JG, Hirschfield GM, Eda S, Eiriksdottir G, Rumley A, Lowe GD, Pepys MB and Gudnason V (2004) C-reactive protein and other circulating markers of inflammation in the prediction of coronary heart disease. New England Journal of Medicine 350, 1387–1397. 15070788 10.1056/NEJMoa032804

[ref13] Danesh J, Kaptoge S, Mann AG, Sarwar N, Wood A, Angleman SB, Wensley F, Higgins JP, Lennon L and Eiriksdottir G (2008) Long-term interleukin-6 levels and subsequent risk of coronary heart disease: two new prospective studies and a systematic review. PLoS Medicine 5, e78. 18399716 10.1371/journal.pmed.0050078 PMC2288623

[ref14] Dantzer R, O'Connor JC, Freund GG, Johnson RW and Kelley KW (2008) From inflammation to sickness and depression: when the immune system subjugates the brain. Nature Reviews Neuroscience 9, 46. 18073775 10.1038/nrn2297 PMC2919277

[ref15] Dickens C, McGowan L, Clark-Carter D and Creed F (2002) Depression in rheumatoid arthritis: a systematic review of the literature with meta-analysis. Psychosomatic Medicine 64, 52–60. 11818586 10.1097/00006842-200201000-00008

[ref16] Dowlati Y, Herrmann N, Swardfager W, Liu H, Sham L, Reim EK and Lanctôt KL (2010) A meta-analysis of cytokines in major depression. Biological Psychiatry 67, 446–457. 20015486 10.1016/j.biopsych.2009.09.033

[ref17] Ekinci O and Ekinci A (2017) The connections among suicidal behavior, lipid profile and low-grade inflammation in patients with major depressive disorder: a specific relationship with the neutrophil-to-lymphocyte ratio. Nordic Journal of Psychiatry 71, 574–580. 28800269 10.1080/08039488.2017.1363285

[ref18] Euteneuer F, Dannehl K, Del Rey A, Engler H, Schedlowski M and Rief W (2017) Immunological effects of behavioral activation with exercise in major depression: an exploratory randomized controlled trial. Translational Psychiatry 7, e1132. 28509904 10.1038/tp.2017.76 PMC5534946

[ref19] Feighner JP, Robins E, Guze SB, Woodruff RA, Winokur G and Munoz R (1972) Diagnostic criteria for use in psychiatric research. Archives of General Psychiatry 26, 57–63. 5009428 10.1001/archpsyc.1972.01750190059011

[ref20] Felger JC, Haroon E, Patel TA, Goldsmith DR, Wommack EC, Woolwine BJ, Le N-A, Feinberg R, Tansey MG and Miller AH (2018) What does plasma CRP tell us about peripheral and central inflammation in depression? Molecular Psychiatry, epub ahead of print 10.1038/s41380-018-0096-3 PMC6291384 29895893

[ref21] Fernandes B, Steiner J, Bernstein H, Dodd S, Pasco J, Dean O, Nardin P, Goncalves C and Berk M (2016) C-reactive protein is increased in schizophrenia but is not altered by antipsychotics: meta-analysis and implications. Molecular Psychiatry 21, 554. 26169974 10.1038/mp.2015.87

[ref22] Ford ES, Giles WH, Mokdad AH and Myers GL (2004) Distribution and correlates of C-reactive protein concentrations among adult US women. Clinical Chemistry 50, 574–581. 14709450 10.1373/clinchem.2003.027359

[ref23] Gallagher D, Kiss A, Lanctot K and Herrmann N (2017) Depression with inflammation: longitudinal analysis of a proposed depressive subtype in community dwelling older adults. International Journal of Geriatric Psychiatry 32, e18–e24. 27911015 10.1002/gps.4645

[ref24] Gimeno D, Kivimäki M, Brunner EJ, Elovainio M, De Vogli R, Steptoe A, Kumari M, Lowe GD, Rumley A and Marmot MG (2009) Associations of C-reactive protein and interleukin-6 with cognitive symptoms of depression: 12-year follow-up of the Whitehall II study. Psychological Medicine 39, 413–423. 18533059 10.1017/S0033291708003723 PMC2788760

[ref25] Goldsmith D, Rapaport M and Miller B (2016) A meta-analysis of blood cytokine network alterations in psychiatric patients: comparisons between schizophrenia, bipolar disorder and depression. Molecular Psychiatry 21, 1696–1709. 26903267 10.1038/mp.2016.3 PMC6056174

[ref26] Haapakoski R, Mathieu J, Ebmeier KP, Alenius H and Kivimäki M (2015) Cumulative meta-analysis of interleukins 6 and 1*β*, tumour necrosis factor *α* and C-reactive protein in patients with major depressive disorder. Brain, Behavior, and Immunity 49, 206–215. 10.1016/j.bbi.2015.06.001 PMC4566946 26065825

[ref27] Hannestad J, DellaGioia N, Gallezot J-D, Lim K, Nabulsi N, Esterlis I, Pittman B, Lee J-Y, O'Connor KC and Pelletier D (2013) The neuroinflammation marker translocator protein is not elevated in individuals with mild-to-moderate depression: a [11C] PBR28 PET study. Brain, Behavior, and Immunity 33, 131–138. 10.1016/j.bbi.2013.06.010 PMC3899398 23850810

[ref28] Hare DL, Toukhsati SR, Johansson P and Jaarsma T (2013) Depression and cardiovascular disease: a clinical review. European Heart Journal 35, 1365–1372. 24282187 10.1093/eurheartj/eht462

[ref29] Harley J, Luty S, Carter J, Mulder R and Joyce P (2010) Elevated C-reactive protein in depression: a predictor of good long-term outcome with antidepressants and poor outcome with psychotherapy. Journal of Psychopharmacology 24, 625. 19282426 10.1177/0269881109102770

[ref30] Haroon E, Fleischer C, Felger J, Chen X, Woolwine B, Patel T, Hu X and Miller A (2016) Conceptual convergence: increased inflammation is associated with increased basal ganglia glutamate in patients with major depression. Molecular Psychiatry 21, 1351. 26754953 10.1038/mp.2015.206 PMC4940313

[ref31] Higgins J and Thompson SG (2002) Quantifying heterogeneity in a meta-analysis. Statistics in Medicine 21, 1539–1558. 12111919 10.1002/sim.1186

[ref32] Horsdal H, Köhler-Forsberg O, Benros M and Gasse C (2017) C-reactive protein and white blood cell levels in schizophrenia, bipolar disorders and depression-associations with mortality and psychiatric outcomes: a population-based study. European Psychiatry 44, 164–172. 28645055 10.1016/j.eurpsy.2017.04.012

[ref33] Howren MB, Lamkin DM and Suls J (2009) Associations of depression with C-reactive protein, IL-1, and IL-6: a meta-analysis. Psychosomatic Medicine 71, 171–186. 19188531 10.1097/PSY.0b013e3181907c1b

[ref34] IL6R Genetics Consortium Emerging Risk Factors Collaboration (2012) Interleukin-6 receptor pathways in coronary heart disease: a collaborative meta-analysis of 82 studies. The Lancet 379, 1205–1213. 10.1016/S0140-6736(11)61931-4 PMC3316940 22421339

[ref35] Interleukin-6 Receptor Mendelian Randomisation Analysis Consortium (2012) The interleukin-6 receptor as a target for prevention of coronary heart disease: a Mendelian randomisation analysis. The Lancet 379, 1214–1224. 10.1016/S0140-6736(12)60110-X PMC3316968 22421340

[ref36] Jha MK, Minhajuddin A, Gadad BS, Greer T, Grannemann B, Soyombo A, Mayes TL, Rush AJ and Trivedi MH (2017) Can C-reactive protein inform antidepressant medication selection in depressed outpatients? Findings from the CO-MED trial. Psychoneuroendocrinology 78, 105–113. 28187400 10.1016/j.psyneuen.2017.01.023 PMC6080717

[ref37] Kappelmann N, Lewis G, Dantzer R, Jones PB and Khandaker GM (2018) Antidepressant activity of anti-cytokine treatment: a systematic review and meta-analysis of clinical trials of chronic inflammatory conditions. Molecular Psychiatry 23, 335. 27752078 10.1038/mp.2016.167 PMC5794896

[ref38] Khandaker GM, Pearson RM, Zammit S, Lewis G and Jones PB (2014) Association of serum interleukin 6 and C-reactive protein in childhood with depression and psychosis in young adult life: a population-based longitudinal study. JAMA Psychiatry 71, 1121–1128. 25133871 10.1001/jamapsychiatry.2014.1332 PMC4561502

[ref39] Khandaker GM, Cousins L, Deakin J, Lennox BR, Yolken R and Jones PB (2015) Inflammation and immunity in schizophrenia: implications for pathophysiology and treatment. The Lancet Psychiatry 2, 258–270. 26359903 10.1016/S2215-0366(14)00122-9 PMC4595998

[ref40] Khandaker GM, Dantzer R and Jones PB (2017) Immunopsychiatry: important facts. Psychological Medicine 47, 2229–2237. 28418288 10.1017/S0033291717000745 PMC5817424

[ref41] Khandaker GM, Oltean BP, Kaser M, Dibben CR, Ramana R, Jadon DR, Dantzer R, Coles AJ, Lewis G and Jones PB (2018) Protocol for the insight study: a randomised controlled trial of single-dose tocilizumab in patients with depression and low-grade inflammation. BMJ Open 8, e025333. 10.1136/bmjopen-2018-025333 PMC6157523 30244217

[ref42] Khandaker GM, Zuber V, Rees JMB, Carvalho L, Mason AM, Foley CN, Gkatzionis A, Jones PB and Burgess S (2019) Shared mechanism between depression and coronary heart disease: findings from Mendelian randomization analysis of a large UK population-based cohort. Molecular Psychiatry, epub ahead of print.

[ref43] Khera A, McGuire DK, Murphy SA, Stanek HG, Das SR, Vongpatanasin W, Wians FH, Grundy SM and de Lemos JA (2005) Race and gender differences in C-reactive protein levels. Journal of the American College of Cardiology 46, 464–469. 16053959 10.1016/j.jacc.2005.04.051

[ref44] Kling MA, Alesci S, Csako G, Costello R, Luckenbaugh DA, Bonne O, Duncko R, Drevets WC, Manji HK and Charney DS (2007) Sustained low-grade pro-inflammatory state in unmedicated, remitted women with major depressive disorder as evidenced by elevated serum levels of the acute phase proteins C-reactive protein and serum amyloid A. Biological Psychiatry 62, 309–313. 17178112 10.1016/j.biopsych.2006.09.033 PMC2546515

[ref45] Köhler O, Benros ME, Nordentoft M, Farkouh ME, Iyengar RL, Mors O and Krogh J (2014) Effect of anti-inflammatory treatment on depression, depressive symptoms, and adverse effects: a systematic review and meta-analysis of randomized clinical trials. JAMA Psychiatry 71, 1381–1391. 25322082 10.1001/jamapsychiatry.2014.1611

[ref46] Ladwig K-H, Marten-Mittag B, Löwel H, Döring A and Koenig W (2005) C-reactive protein, depressed mood, and the prediction of coronary heart disease in initially healthy men: results from the MONICA–KORA Augsburg Cohort Study 1984–1998. European Heart Journal 26, 2537–2542. 16126719 10.1093/eurheartj/ehi456

[ref47] Lanquillon S, Krieg J-C, Bening-Abu-Shach U and Vedder H (2000) Cytokine production and treatment response in major depressive disorder. Neuropsychopharmacology 22, 370–379. 10700656 10.1016/S0893-133X(99)00134-7

[ref48] Legros S, Mendlewicz J and Wybran J (1985) Immunoglobulins, autoantibodies and other serum protein fractions in psychiatric disorders. European Archives of Psychiatry and Neurological Sciences 235, 9–11. 3876216 10.1007/BF00380962

[ref49] Li Y, Zhong X, Cheng G, Zhao C, Zhang L, Hong Y, Wan Q, He R and Wang Z (2017) Hs-CRP and all-cause, cardiovascular, and cancer mortality risk: a meta-analysis. Atherosclerosis 259, 75–82. 28327451 10.1016/j.atherosclerosis.2017.02.003

[ref50] Lim GY, Tam WW, Lu Y, Ho CS, Zhang MW and Ho RC (2018) Prevalence of depression in the community from 30 countries between 1994 and 2014. Scientific Reports 8, 2861. 29434331 10.1038/s41598-018-21243-x PMC5809481

[ref51] Liukkonen T, Silvennoinen-Kassinen S, Jokelainen J, Räsänen P, Leinonen M, Meyer-Rochow VB and Timonen M (2006) The association between C-reactive protein levels and depression: results from the northern Finland 1966 birth cohort study. Biological Psychiatry 60, 825–830. 16616729 10.1016/j.biopsych.2006.02.016

[ref52] Ma Y, Chiriboga DE, Pagoto SL, Rosal MC, Li W, Merriam PA, Hébert JR, Whited MC and Ockene IS (2011) Association between depression and C-reactive protein. Cardiology Research and Practice 2011, 286509. 10.4061/2011/286509 PMC3014664 21234098

[ref53] Maes M, Bosmans E, De Jongh R, Kenis G, Vandoolaeghe E and Neels H (1997) Increased serum IL-6 and IL-1 receptor antagonist concentrations in major depression and treatment resistant depression. Cytokine 9, 853–858. 9367546 10.1006/cyto.1997.0238

[ref54] Miller BJ, Buckley P, Seabolt W, Mellor A and Kirkpatrick B (2011) Meta-analysis of cytokine alterations in schizophrenia: clinical status and antipsychotic effects. Biological Psychiatry 70, 663–671. 21641581 10.1016/j.biopsych.2011.04.013 PMC4071300

[ref55] Müller N, Schwarz M, Dehning S, Douhe A, Cerovecki A, Goldstein-Müller B, Spellmann I, Hetzel G, Maino K and Kleindienst N (2006) The cyclooxygenase-2 inhibitor celecoxib has therapeutic effects in major depression: results of a double-blind, randomized, placebo controlled, add-on pilot study to reboxetine. Molecular Psychiatry 11, 680. 16491133 10.1038/sj.mp.4001805

[ref56] Naghashpour M, Amani R, Nematpour S and Haghighizadeh MH (2011) Riboflavin status and its association with serum hs-CRP levels among clinical nurses with depression. Journal of the American College of Nutrition 30, 340–347. 22081620 10.1080/07315724.2011.10719977

[ref57] Nicholson A, Kuper H and Hemingway H (2006) Depression as an aetiologic and prognostic factor in coronary heart disease: a meta-analysis of 6362 events among 146 538 participants in 54 observational studies. European Heart Journal 27, 2763–2774. 17082208 10.1093/eurheartj/ehl338

[ref58] Nilsson K, Gustafson L and Hultberg B (2008) C-reactive protein: vascular risk marker in elderly patients with mental illness. Dementia and Geriatric Cognitive Disorders 26, 251–256. 18841009 10.1159/000160957

[ref59] O'brien SM, Scott LV and Dinan TG (2006) Antidepressant therapy and C-reactive protein levels. The British Journal of Psychiatry 188, 449–452. 16648531 10.1192/bjp.bp.105.011015

[ref60] Osimo E, Baxter L, Jones P and Khandaker G (2018 *a*) Prevalence of low-grade inflammation in depression: a review and meta-analysis of CRP data. PROSPERO. Available at http://www.crd.york.ac.uk/PROSPERO/display_record.php?ID=CRD42018106640. 10.1017/S0033291719001454 PMC6712955 31258105

[ref61] Osimo EF, Cardinal RN, Jones PB and Khandaker GM (2018 *b*) Prevalence and correlates of low-grade systemic inflammation in adult psychiatric inpatients: an electronic health record-based study. Psychoneuroendocrinology 91, 226–234. 29544672 10.1016/j.psyneuen.2018.02.031 PMC5910056

[ref62] Park S, Joo YH, McIntyre RS and Kim B (2014) Metabolic syndrome and elevated C-reactive protein levels in elderly patients with newly diagnosed depression. Psychosomatics 55, 640–649. 24629898 10.1016/j.psym.2013.12.010

[ref63] Parloff MB, Kelman HC and Frank JD (1954) Comfort, effectiveness, and self-awareness as criteria of improvement in psychotherapy. American Journal of Psychiatry 111, 343–352. 13197596 10.1176/ajp.111.5.343

[ref64] Pearson TA, Mensah GA, Alexander RW, Anderson JL, Cannon III RO, Criqui M, Fadl YY, Fortmann SP, Hong Y and Myers GL (2003) Markers of inflammation and cardiovascular disease: application to clinical and public health practice: a statement for healthcare professionals from the Centers for Disease Control and Prevention and the American Heart Association. Circulation 107, 499–511. 12551878 10.1161/01.cir.0000052939.59093.45

[ref65] Penninx BW, Kritchevsky SB, Yaffe K, Newman AB, Simonsick EM, Rubin S, Ferrucci L, Harris T and Pahor M (2003) Inflammatory markers and depressed mood in older persons: results from the health, aging and body composition study. Biological Psychiatry 54, 566–572. 12946885 10.1016/s0006-3223(02)01811-5

[ref66] Porcu M, Urbano MR, Verri WA Jr, Barbosa DS, Baracat M, Vargas HO, Machado RCBR, Pescim RR and Nunes SOV (2018) Effects of adjunctive *N*-acetylcysteine on depressive symptoms: modulation by baseline high-sensitivity C-reactive protein. Psychiatry Research 263, 268–274. 29605103 10.1016/j.psychres.2018.02.056

[ref67] Pradhan AD, Manson JE, Rifai N, Buring JE and Ridker PM (2001) C-reactive protein, interleukin 6, and risk of developing type 2 diabetes mellitus. JAMA 286, 327–334. 11466099 10.1001/jama.286.3.327

[ref68] Pradhan AD, Manson JE, Rossouw JE, Siscovick DS, Mouton CP, Rifai N, Wallace RB, Jackson RD, Pettinger MB and Ridker PM (2002) Inflammatory biomarkers, hormone replacement therapy, and incident coronary heart disease: prospective analysis from the Women's Health Initiative observational study. JAMA 288, 980–987. 12190368 10.1001/jama.288.8.980

[ref69] Raison CL, Rutherford RE, Woolwine BJ, Shuo C, Schettler P, Drake DF, Haroon E and Miller AH (2013) A randomized controlled trial of the tumor necrosis factor antagonist infliximab for treatment-resistant depression: the role of baseline inflammatory biomarkers. JAMA Psychiatry 70, 31–41. 22945416 10.1001/2013.jamapsychiatry.4 PMC4015348

[ref70] Rapaport MH, Nierenberg AA, Schettler PJ, Kinkead B, Cardoos A, Walker R and Mischoulon D (2016) Inflammation as a predictive biomarker for response to omega-3 fatty acids in major depressive disorder: a proof-of-concept study. Molecular Psychiatry 21, 71. 25802980 10.1038/mp.2015.22 PMC4581883

[ref71] R Core Team (2017). R: A Language and Environment for Statistical Computing [Software]. Vienna, Austria: R Foundation for Statistical Computing.

[ref72] Ridker PM (2003) Clinical application of C-reactive protein for cardiovascular disease detection and prevention. Circulation 107, 363–369. 12551853 10.1161/01.cir.0000053730.47739.3c

[ref73] Schmidt R, Schmidt H, Curb JD, Masaki K, White LR and Launer LJ (2002) Early inflammation and dementia: a 25-year follow-up of the Honolulu-Asia Aging Study. Annals of Neurology 52, 168–174. 12210786 10.1002/ana.10265

[ref74] Schwarzer G (2007) Meta’: an R package for meta-analysis: R news, 7, 40–45.

[ref75] Shanahan L, Copeland WE, Worthman CM, Angold A and Costello EJ (2013) Children with both asthma and depression are at risk for heightened inflammation. The Journal of Pediatrics 163, 1443–1447. 23919906 10.1016/j.jpeds.2013.06.046 PMC3967500

[ref76] Shibata M, Ohara T, Yoshida D, Hata J, Mukai N, Kawano H, Kanba S, Kitazono T and Ninomiya T (2018) Association between the ratio of serum arachidonic acid to eicosapentaenoic acid and the presence of depressive symptoms in a general Japanese population: the Hisayama Study. Journal of Affective Disorders 237, 73–79. 29787929 10.1016/j.jad.2018.05.004

[ref77] Shin Y-C, Jung C-H, Kim H-J, Kim E-J and Lim S-W (2016) The associations among vitamin D deficiency, C-reactive protein, and depressive symptoms. Journal of Psychosomatic Research 90, 98–104. 27772566 10.1016/j.jpsychores.2016.10.001

[ref78] Sluzewska A, Sobieska M and Rybakowski J (1997) Changes in acute-phase proteins during lithium potentiation of antidepressants in refractory depression. Neuropsychobiology 35, 123–127. 9170116 10.1159/000119332

[ref79] Stang A (2010) Critical evaluation of the Newcastle-Ottawa scale for the assessment of the quality of nonrandomized studies in meta-analyses. European Journal of Epidemiology 25, 603–605. 20652370 10.1007/s10654-010-9491-z

[ref80] Sun Y, Wang D, Salvadore G, Hsu B, Curran M, Casper C, Vermeulen J, Kent JM, Singh J and Drevets WC (2017) The effects of interleukin-6 neutralizing antibodies on symptoms of depressed mood and anhedonia in patients with rheumatoid arthritis and multicentric Castleman's disease. Brain, Behavior, and Immunity 66, 156–164. 10.1016/j.bbi.2017.06.014 28676350

[ref81] Sung K-C, Ryu S, Chang Y, Byrne CD and Kim SH (2014) C-reactive protein and risk of cardiovascular and all-cause mortality in 268 803 East Asians. European Heart Journal 35, 1809–1816. 24569028 10.1093/eurheartj/ehu059

[ref82] Uher R, Tansey KE, Dew T, Maier W, Mors O, Hauser J, Dernovsek MZ, Henigsberg N, Souery D and Farmer A (2014) An inflammatory biomarker as a differential predictor of outcome of depression treatment with escitalopram and nortriptyline. American Journal of Psychiatry 171, 1278–1286. 25017001 10.1176/appi.ajp.2014.14010094

[ref83] Urbanek S and Horner J (2015) Cairo: R graphics device using Cairo graphics library for creating high-quality bitmap (PNG, JPEG, TIFF), vector (PDF, SVG, PostScript) and display (X11 and Win32) output [Software]. CRAN. R-project.

[ref84] Visser M, Bouter LM, McQuillan GM, Wener MH and Harris TB (1999) Elevated C-reactive protein levels in overweight and obese adults. JAMA 282, 2131–2135. 10591334 10.1001/jama.282.22.2131

[ref85] von Känel R, Hepp U, Kraemer B, Traber R, Keel M, Mica L and Schnyder U (2007) Evidence for low-grade systemic proinflammatory activity in patients with posttraumatic stress disorder. Journal of Psychiatric Research 41, 744–752. 16901505 10.1016/j.jpsychires.2006.06.009

[ref86] von Zerssen D and Cording C (1978) The measurement of change in endogenous affective disorders. Archiv für Psychiatrie und Nervenkrankheiten 226, 95–112. 570027 10.1007/BF00345946

[ref87] Wei L, Du Y, Wu W, Fu X and Xia Q (2018) Elevation of plasma neutrophil gelatinase-associated lipocalin (NGAL) levels in schizophrenia patients. Journal of Affective Disorders 226, 307–312. 29028592 10.1016/j.jad.2017.10.002

[ref88] Wium-Andersen MK, Ørsted DD, Nielsen SF and Nordestgaard BG (2013) Elevated C-reactive protein levels, psychological distress, and depression in 73 131 individuals. JAMA Psychiatry 70, 176–184. 23266538 10.1001/2013.jamapsychiatry.102

[ref89] Wium-Andersen MK, Ørsted DD and Nordestgaard BG (2014) Elevated C-reactive protein, depression, somatic diseases, and all-cause mortality: a Mendelian randomization study. Biological Psychiatry 76, 249–257. 24246360 10.1016/j.biopsych.2013.10.009

[ref90] Wysokiński A, Margulska A, Strzelecki D and Kłoszewska I (2015) Levels of C-reactive protein (CRP) in patients with schizophrenia, unipolar depression and bipolar disorder. Nordic Journal of Psychiatry 69, 346–353. 25495587 10.3109/08039488.2014.984755

[ref91] Zacho J, Tybjærg-Hansen A and Nordestgaard BG (2010) C-reactive protein and all-cause mortality – the Copenhagen city heart study. European Heart Journal 31, 1624–1632. 20423919 10.1093/eurheartj/ehq103

[ref92] Zalli A, Jovanova O, Hoogendijk W, Tiemeier H and Carvalho L (2016) Low-grade inflammation predicts persistence of depressive symptoms. Psychopharmacology 233, 1669–1678. 25877654 10.1007/s00213-015-3919-9 PMC4828485

