# Supplementary material for: Prevalence of low-grade inflammation in depression: a systematic review and meta-analysis of CRP levels
Source: Psychol Med. 2019 Jul 1;49(12):1958–70. doi: 10.1017/S0033291719001454 (PMC6712955; doi:10.1017/S0033291719001454)
Supplement: Supplementary file 1 [file S0033291719001454sup001.docx]

Osimo *et al*. Prevalence of Low-grade Inflammation in Depression: a systematic review and meta-analysis of CRP levels

Supplementary material

# Table of contents

Table of contents 1

Supplementary figures 2

Supplementary methods 19

Data Extraction: 19

Quality assessment of studies included in meta-analysis. 19

Supplementary results 20

Supplementary tables 21

Supplementary Table 1: Quality Assessment of Included Studies 21

Supplementary Table 2: Matching criteria for case-controls studies defining inflammation as CRP >3mg/L 23

Supplementary references 24

# **Supplementary figures**

**Supplementary Figure 1: PRISMA diagram of literature search and study selection.**


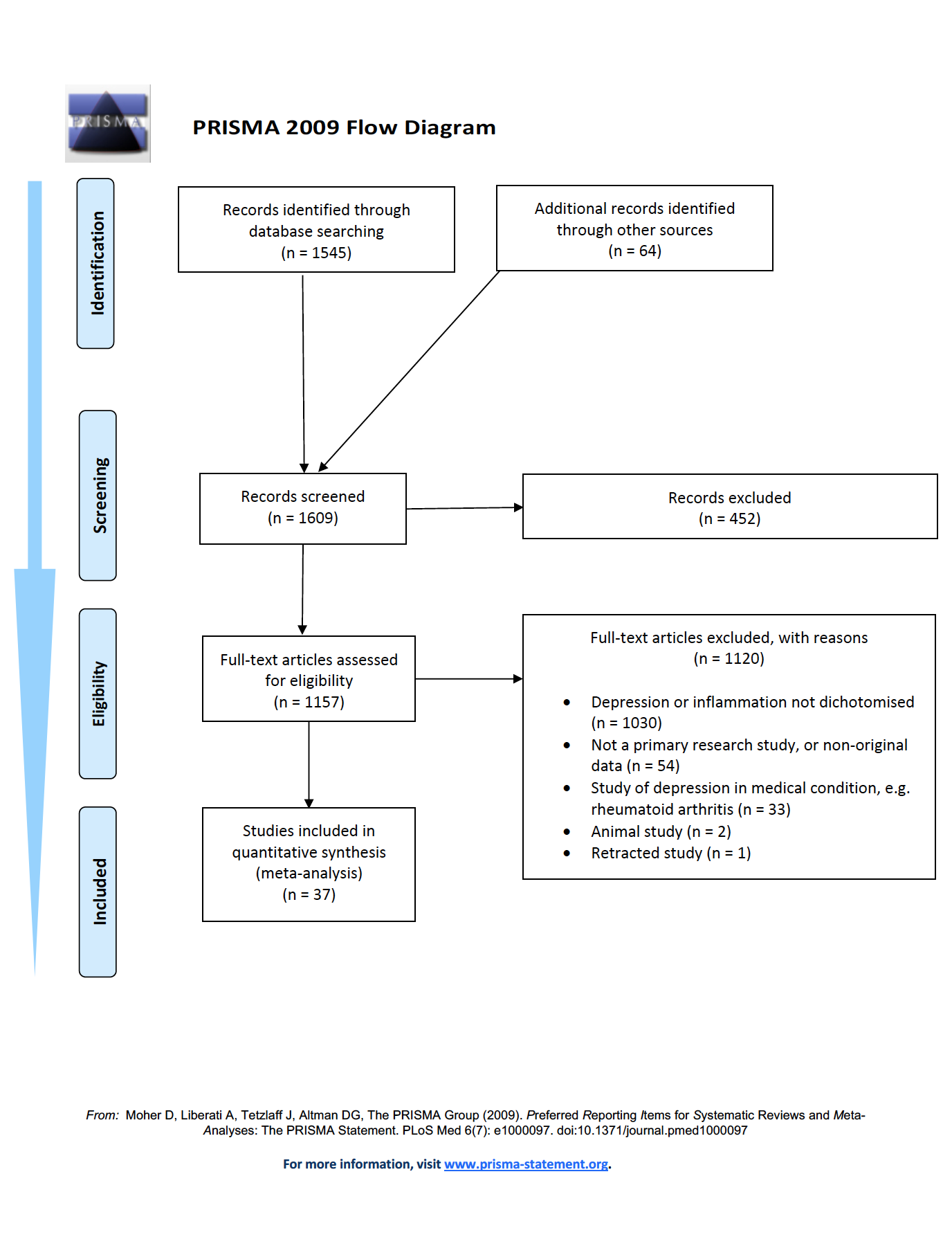


**Supplementary Figure 2: Prevalence of Low-grade Inflammation (CRP >3 mg/L) in Depressed Patients, Excluding Poor Quality Studies**
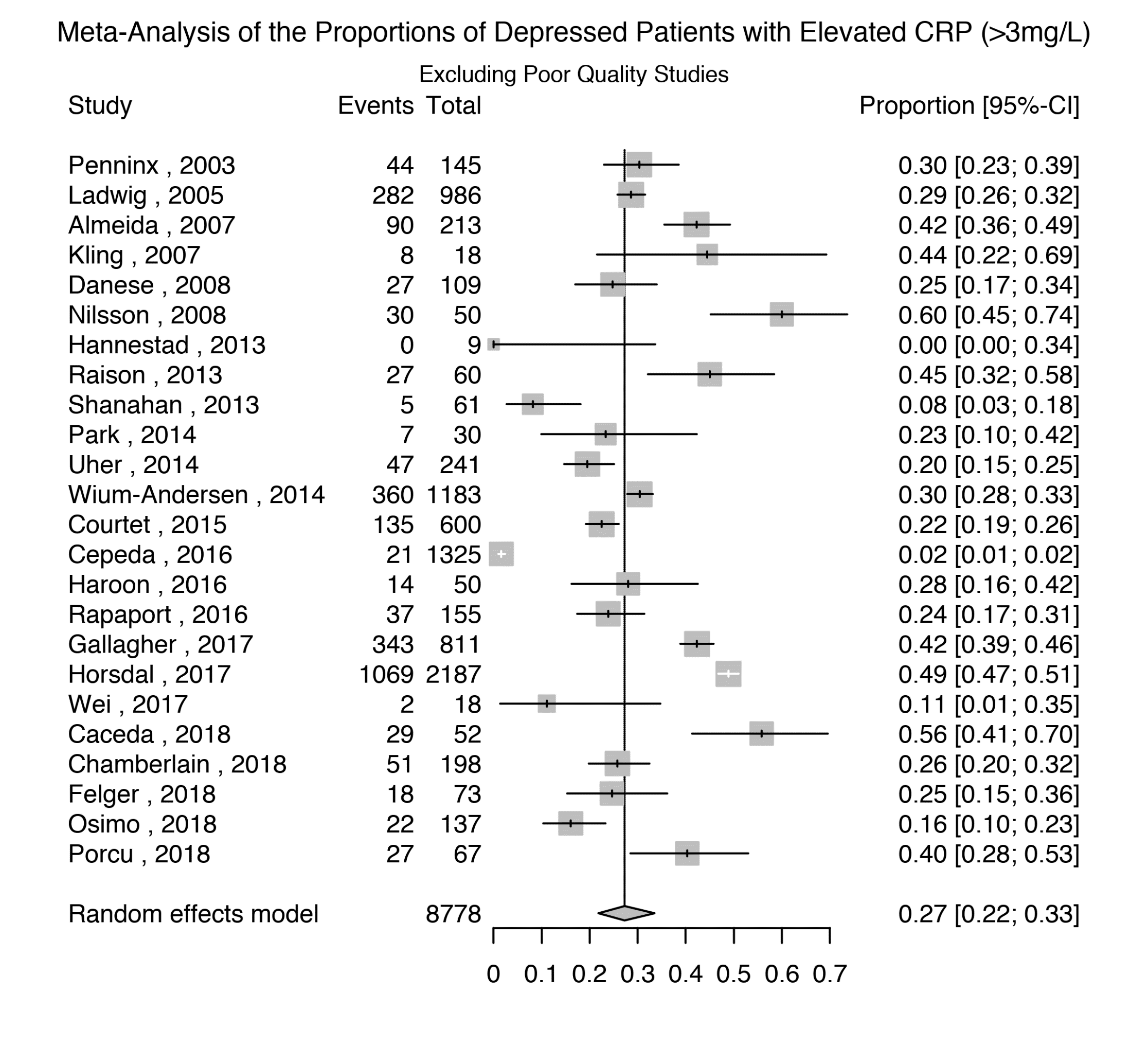


**Supplementary Figure 3: Prevalence of Low-grade Inflammation (CRP >3 mg/L) in Depressed Patients, excluding studies where depression was not active in all patients**.**
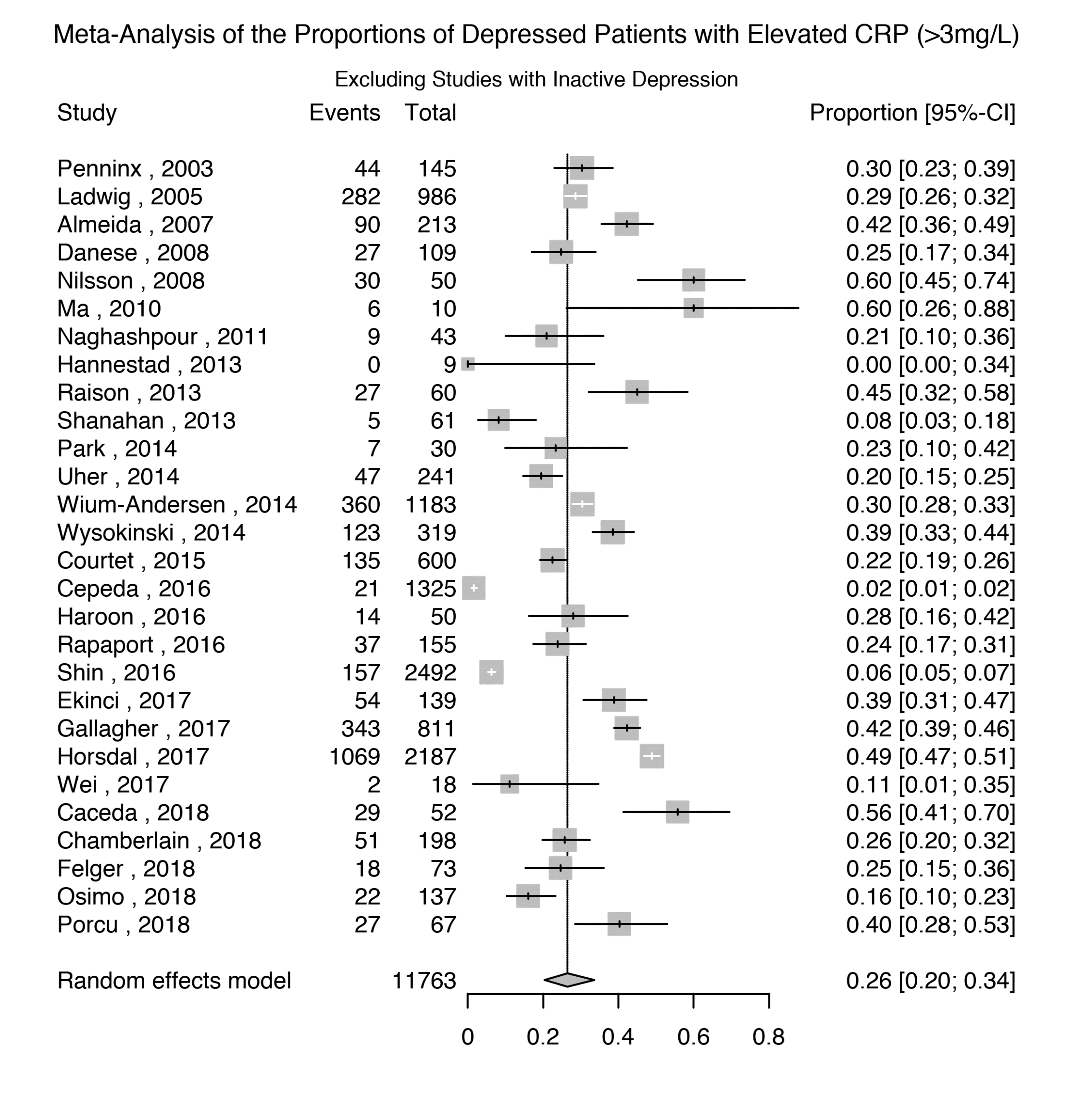
**

**Supplementary Figure 4: Prevalence of Low-grade Inflammation (CRP >3 mg/L) in Depressed Patients, excluding subjects with Suspected Infection (CRP>10 mg/L)**.
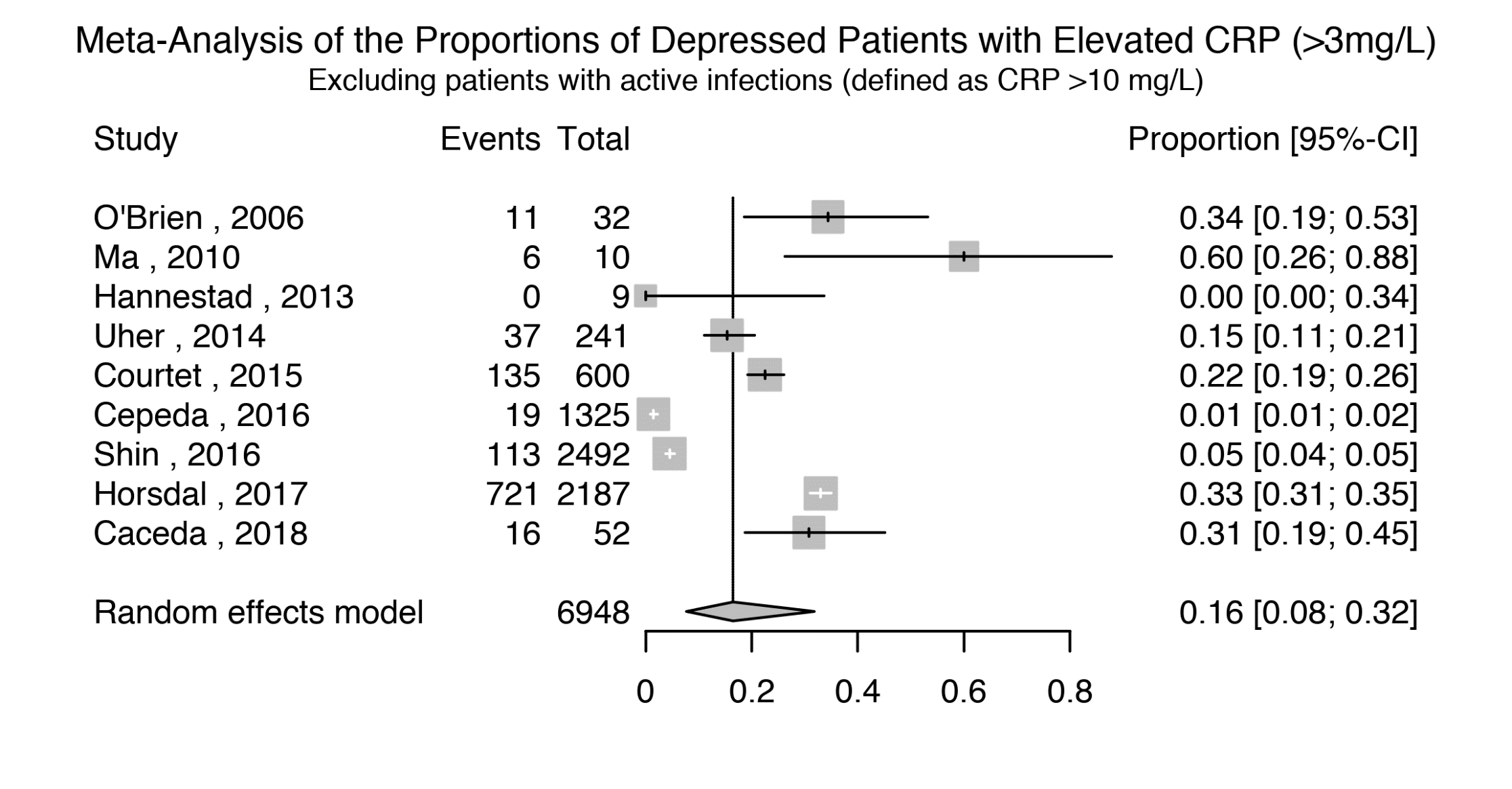


**Supplementary Figure 5: Funnel Plot for Studies Assessing Low-grade Inflammation (CRP >3 mg/L).**
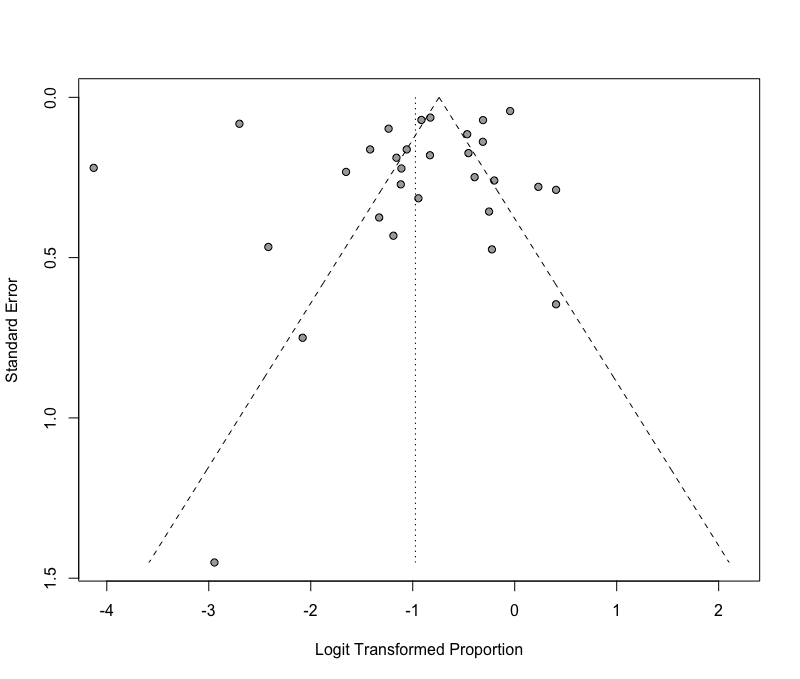


**Supplementary Figure 6: Low-grade Inflammation (CRP >3 mg/L) in Non-depressed Controls Matched to Depressed Patients.**
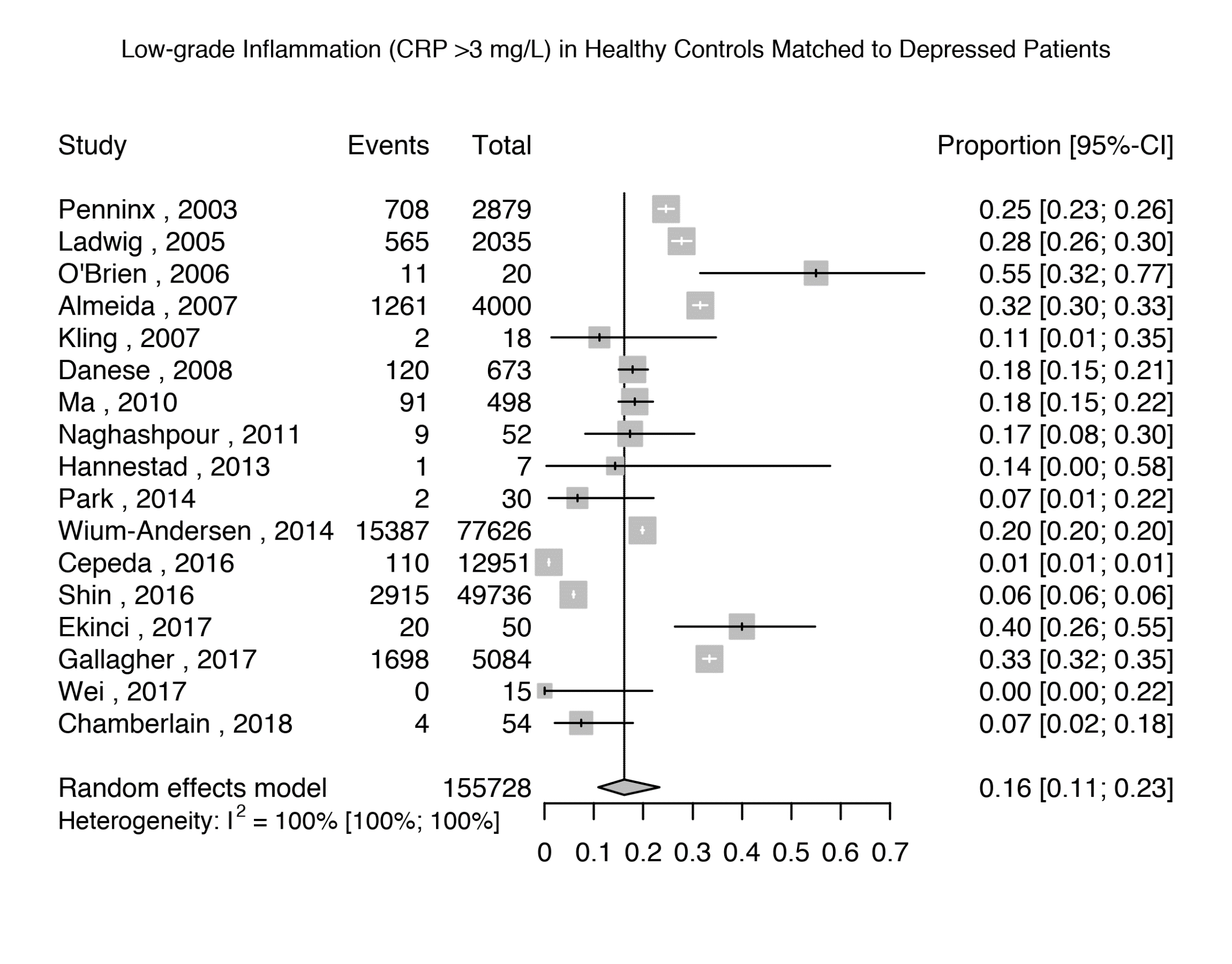


**Supplementary Figure 7: Low-grade Inflammation (CRP >3 mg/L) in Depressed Patients Matched to Non-depressed Controls**.
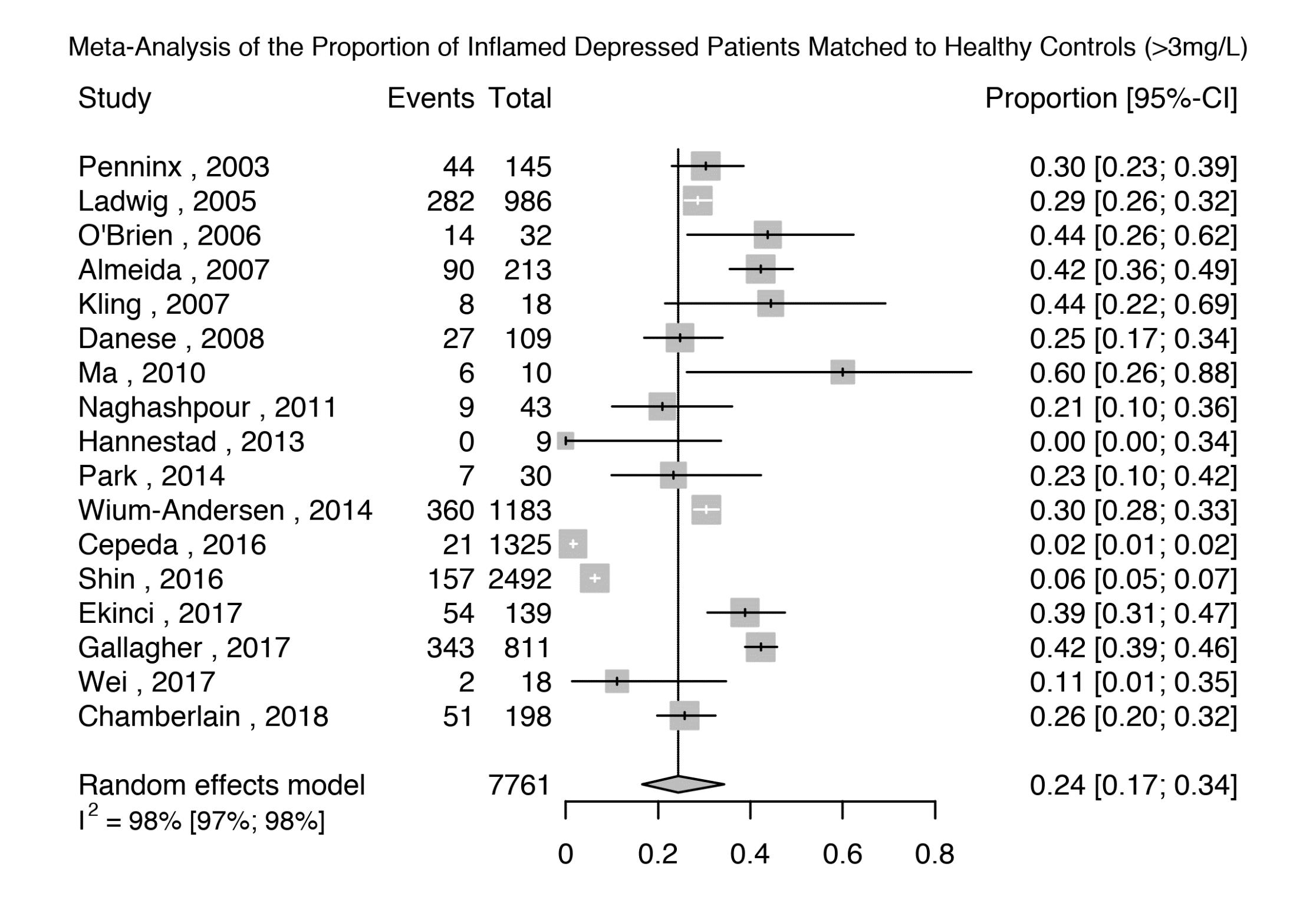


**Supplementary Figure 8: Low-grade Inflammation (CRP >3 mg/L) in Non-depressed Controls Matched to Depressed Patients – excluding patients with suspected infection.
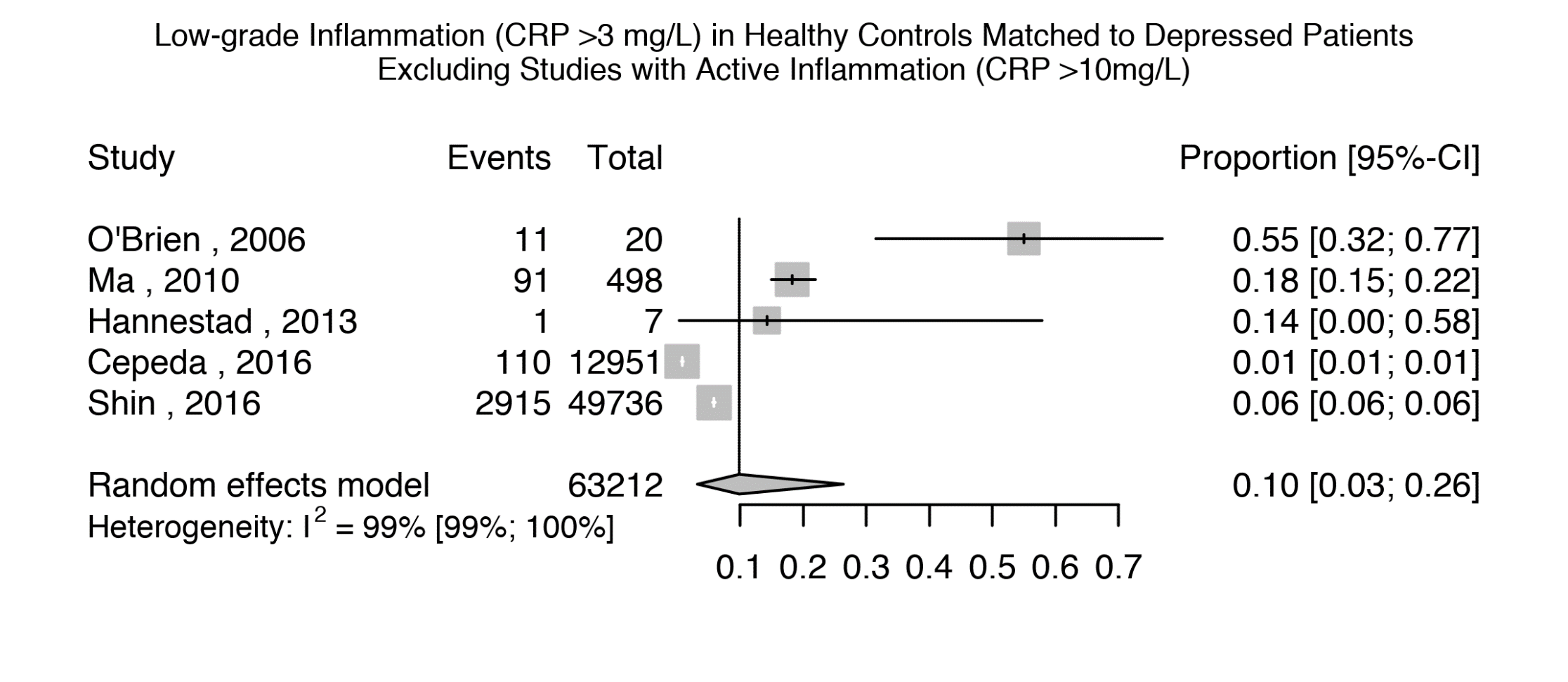
**

**Supplementary Figure 9: Low-grade Inflammation (CRP >3 mg/L) in Depressed Patients Matched to Non-depressed Controls – excluding studies with suspected infection**.
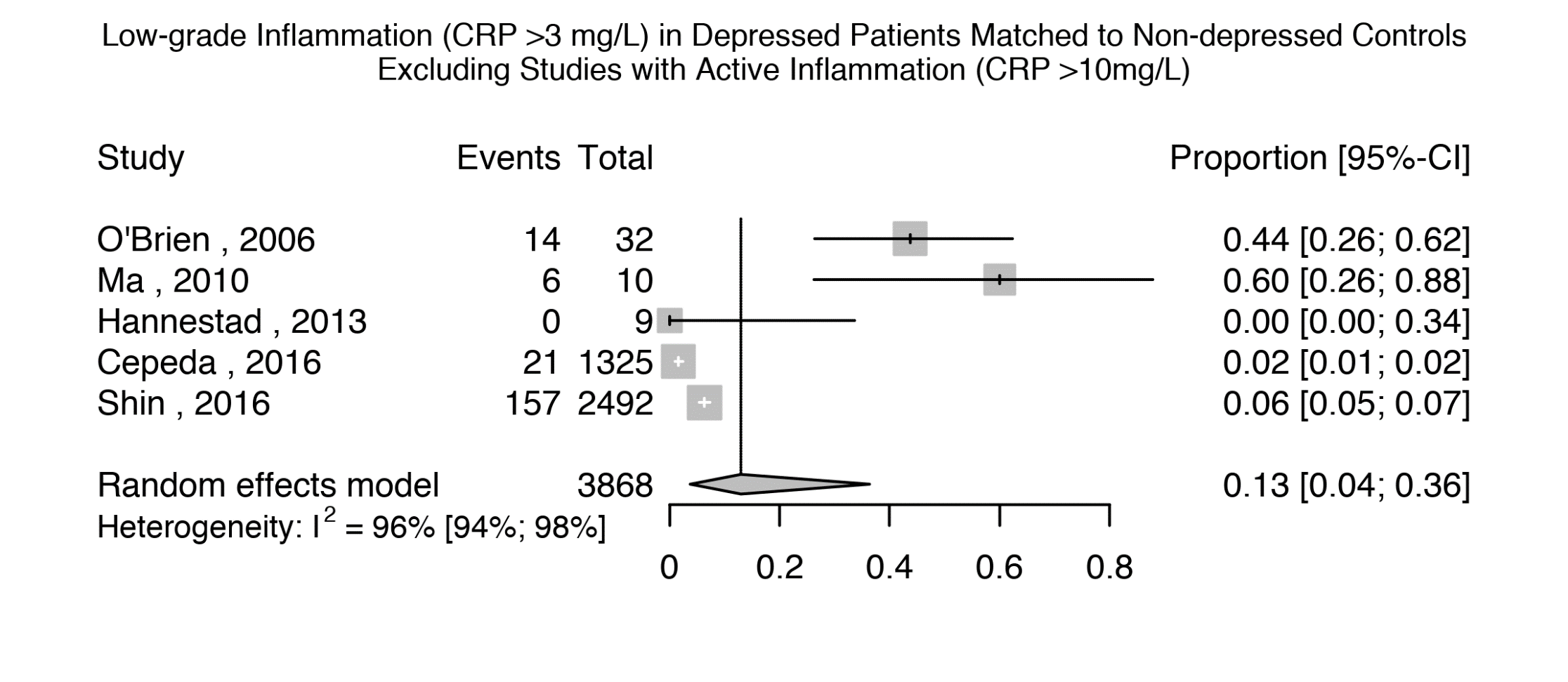


**Supplementary Figure 10: Odds Ratio for Inflammation (CRP >3mg/L) in Depressed Patients Compared with Matched Controls, studies excluding very high levels of inflammation (>10mg/L)**
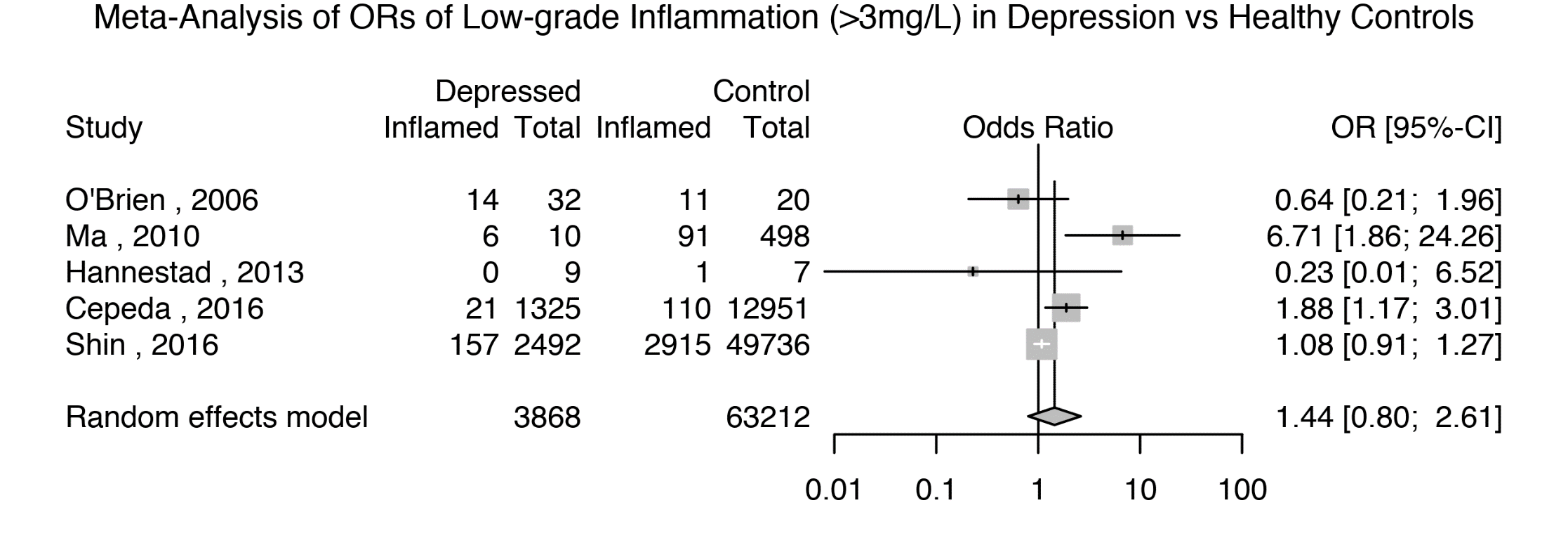


**Supplementary Figure 11: Odds Ratio for Inflammation (CRP >3mg/L) in Depressed Patients Compared with Matched Controls, Excluding Poor Quality Studies
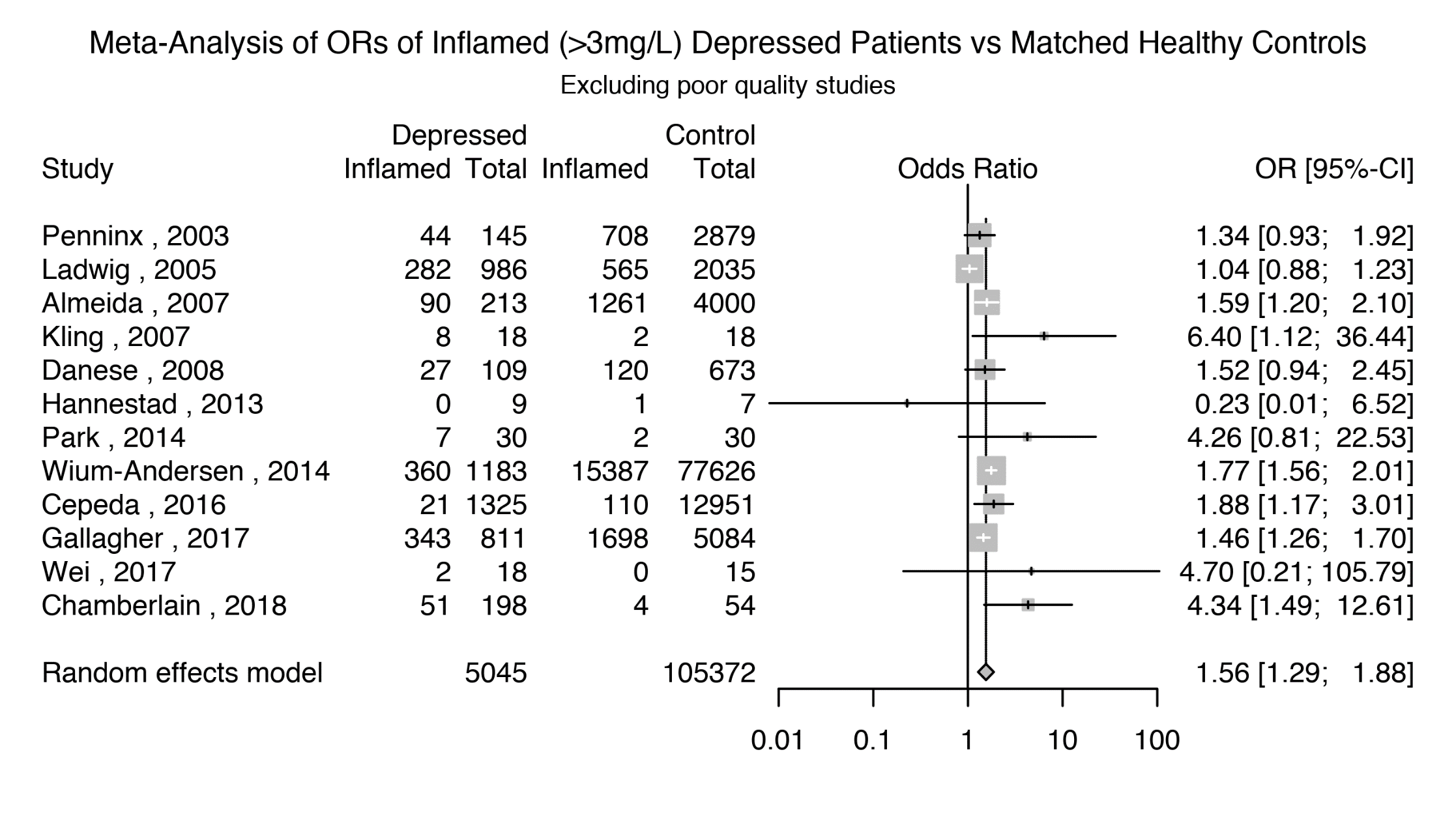
**

**Supplementary Figure 12: Odds Ratio for Inflammation (CRP >3mg/L) in Depressed Patients Compared with Matched Controls, only including BMI-matched studies.**
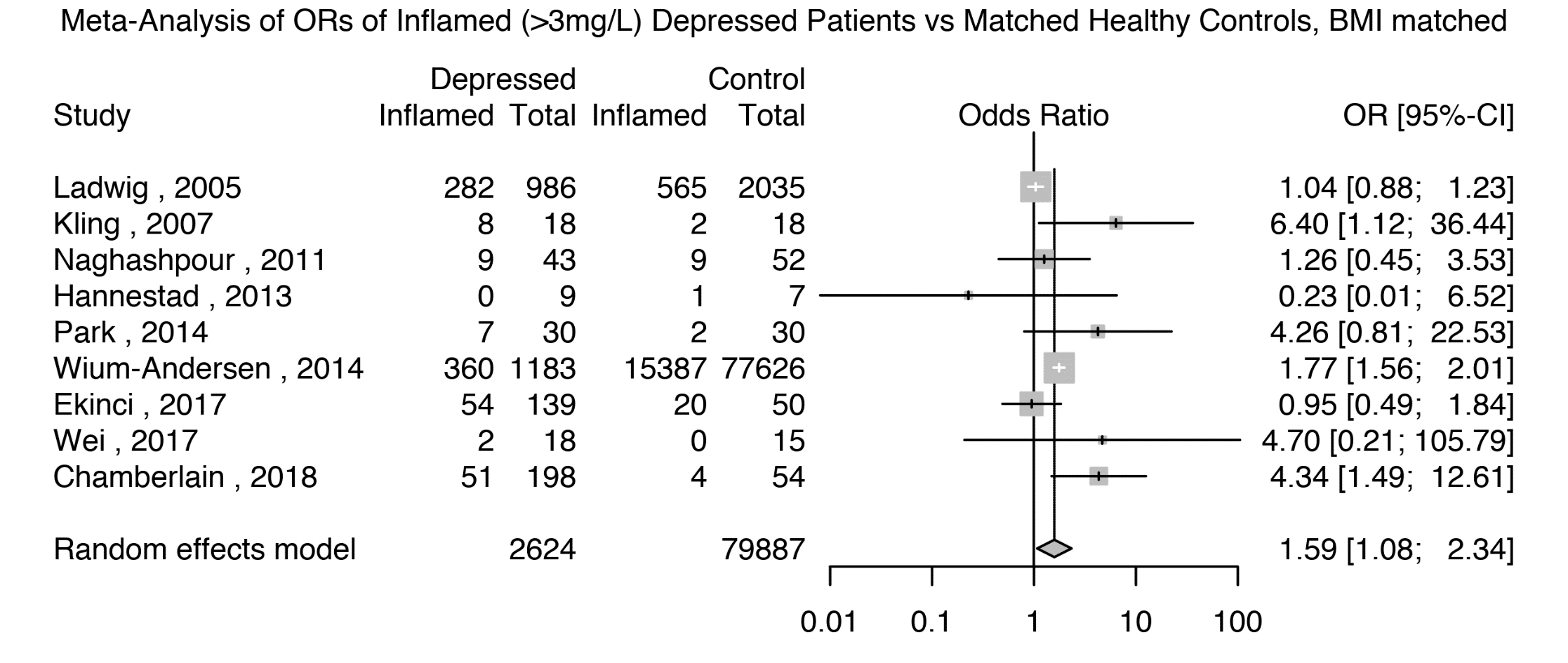


**Supplementary Figure 13: Odds Ratio for Inflammation (CRP >3mg/L) in Depressed Patients Compared with Matched Controls, excluding studies where depression was not active.
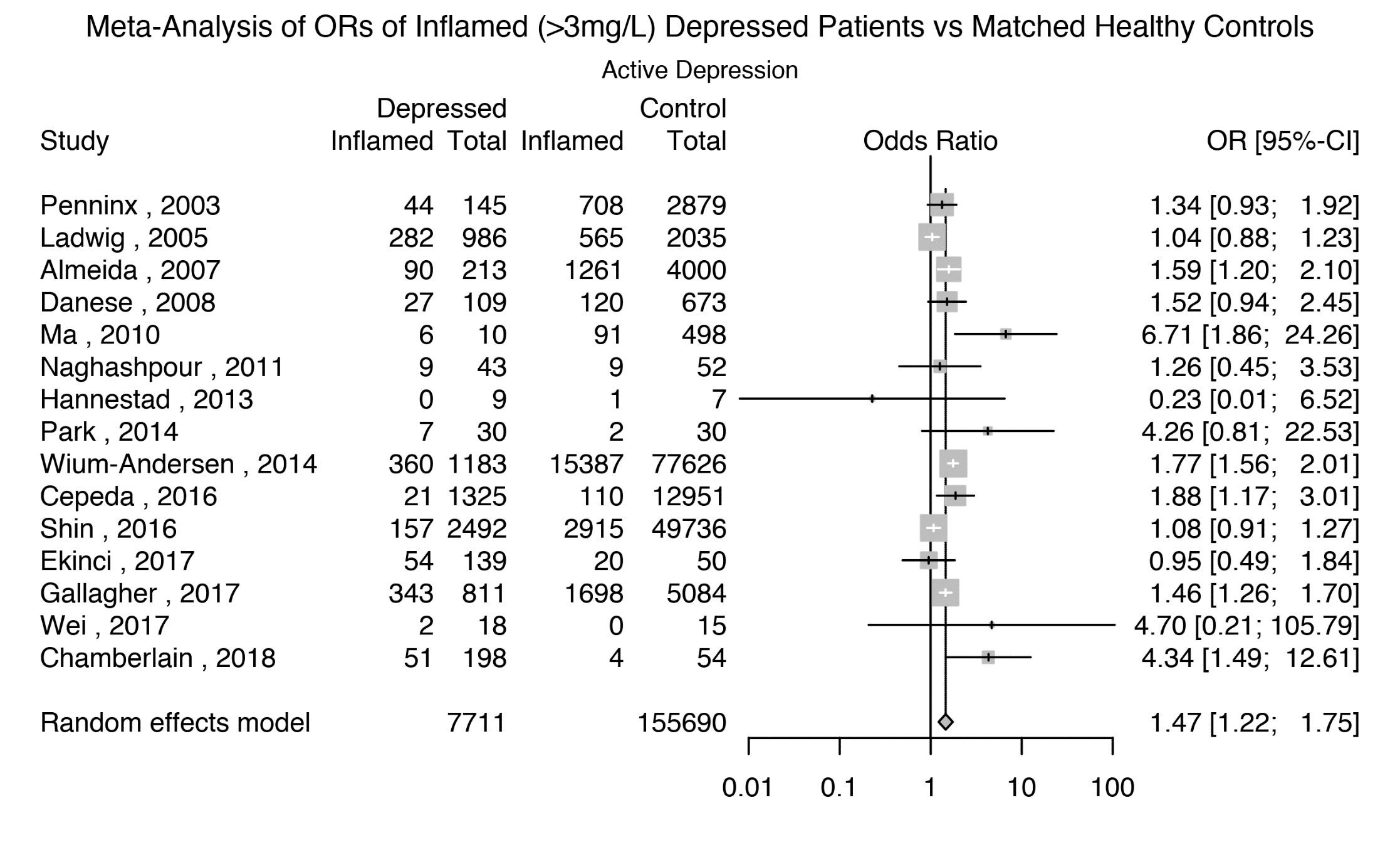
**

**Supplementary Figure 14: Prevalence of Elevated CRP levels (>1 mg/L) in Depressed Patients, Excluding Poor Quality Studies.**
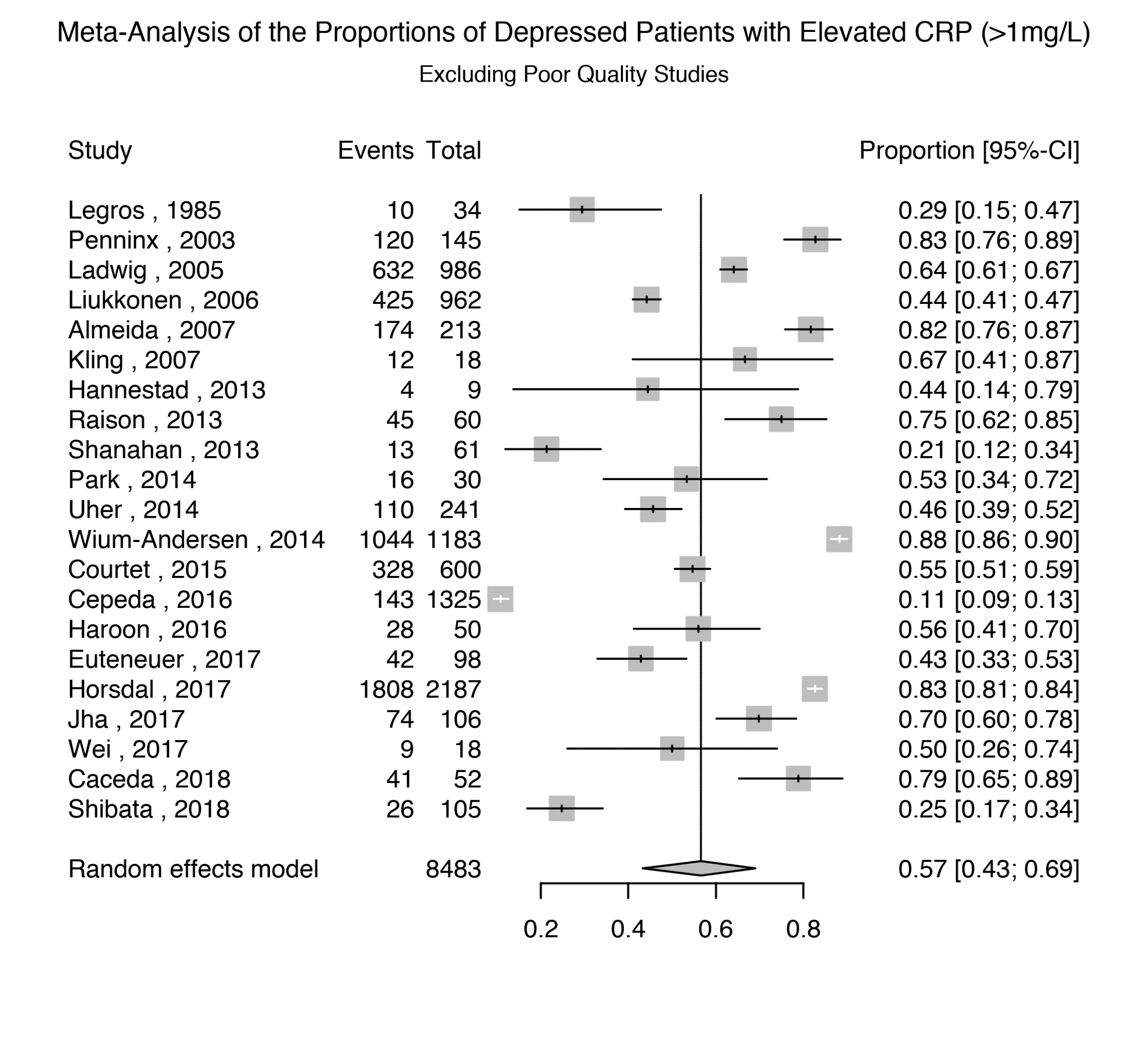


**Supplementary Figure 15: Prevalence of Elevated CRP levels (>1 mg/L) in Depressed Patients, Excluding Studies with Inactive Depression.
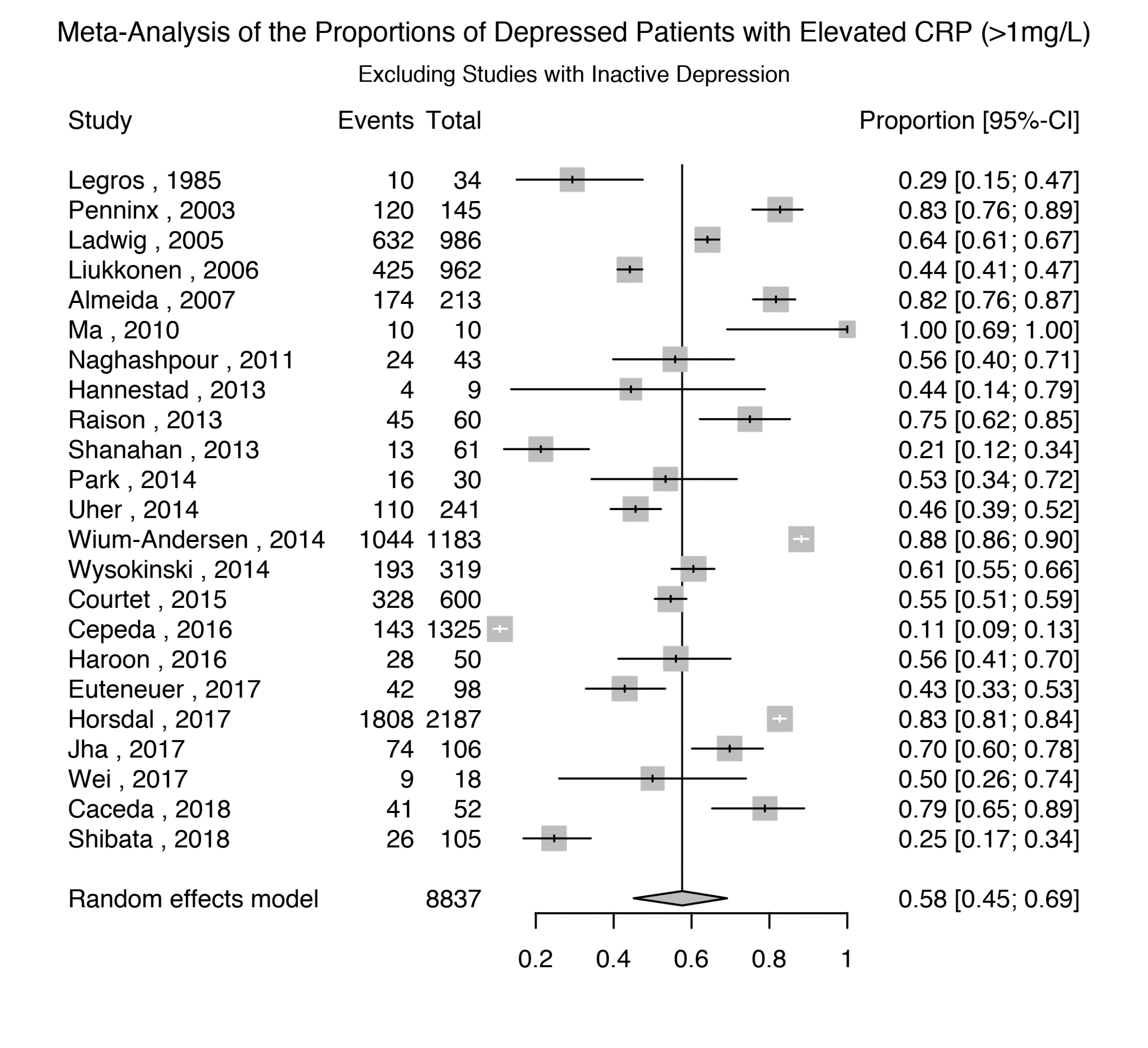
**

**Supplementary Figure 16: Prevalence of Elevated CRP levels (>1 mg/L) in Depressed Patients, Excluding Subjects with Active Infections (CRP>10mg/L).**
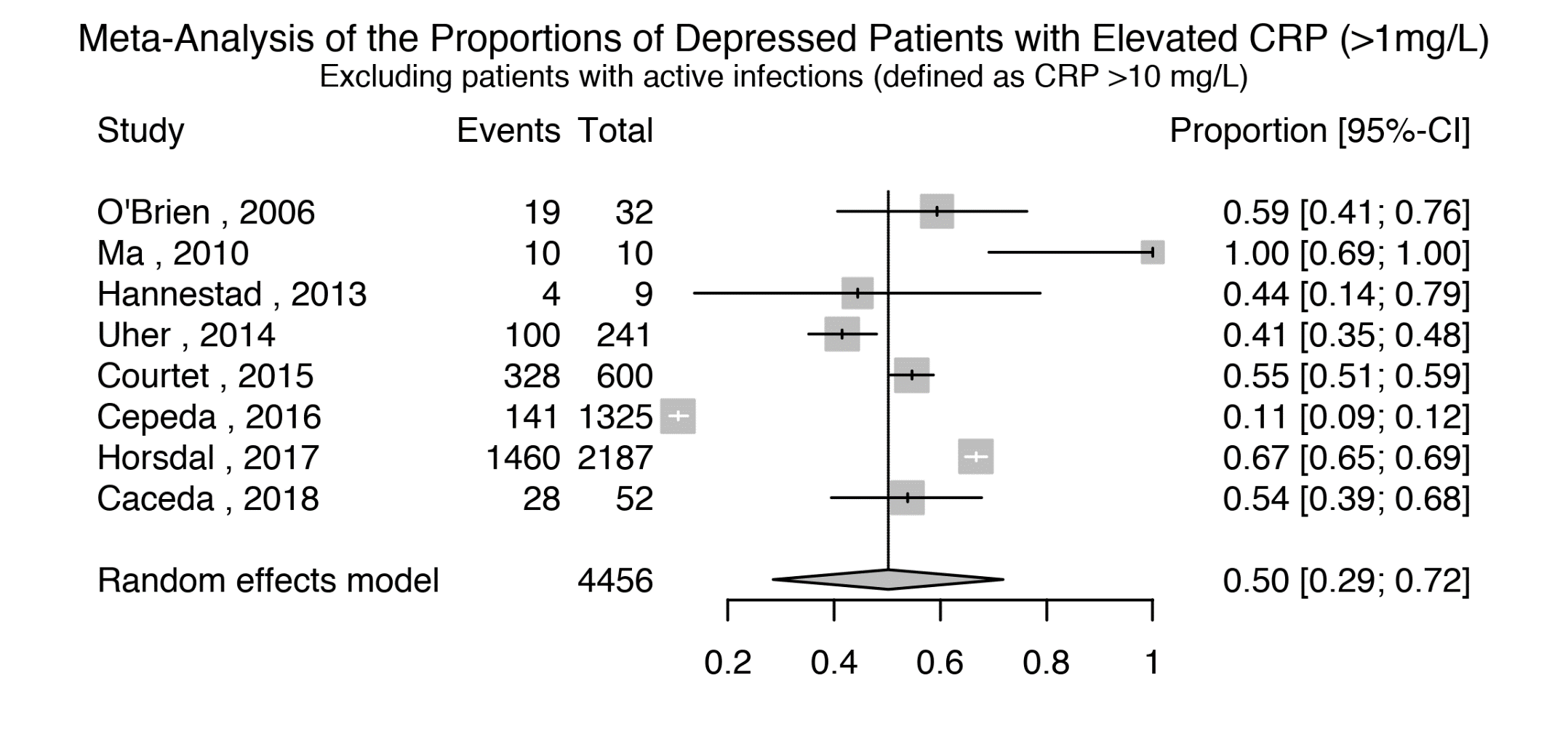


**Supplementary Figure 17: Funnel Plot for Studies Assessing Elevated CRP levels (>1 mg/L).**
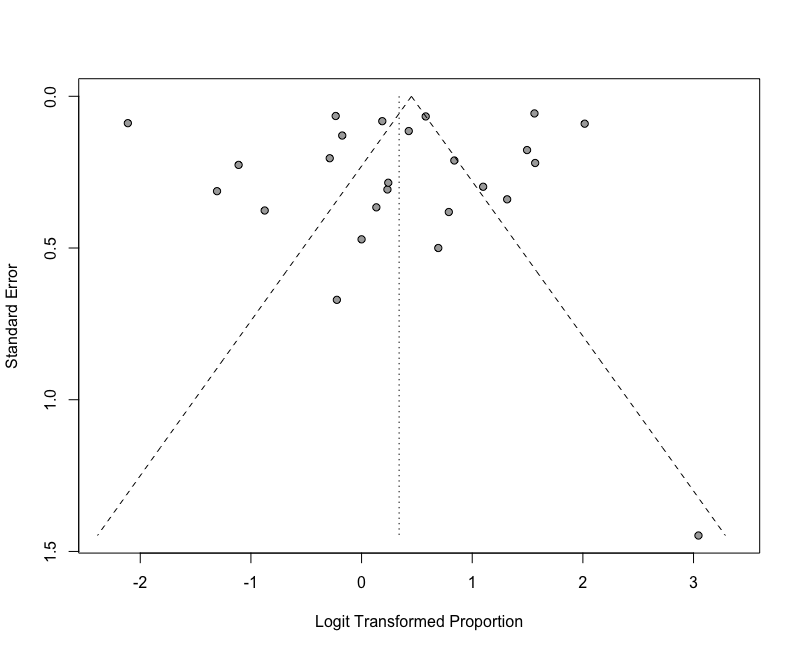


**Supplementary Figure 18: Elevated CRP levels (>1 mg/L) in Non-depressed Controls.**
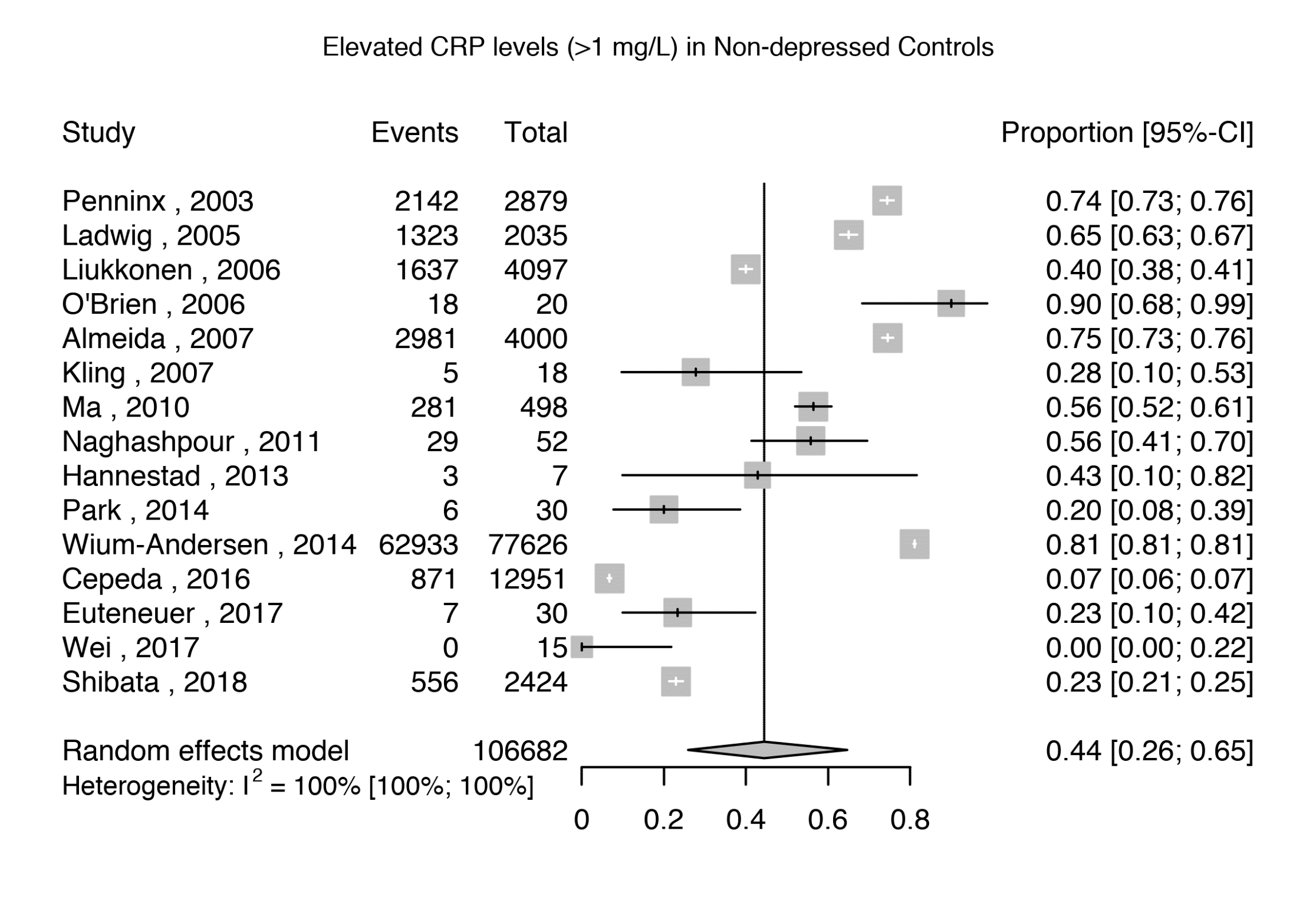


**Supplementary Figure 19: Elevated CRP levels (>1 mg/L) in Depressed Patients Matched to Non-depressed Controls**.
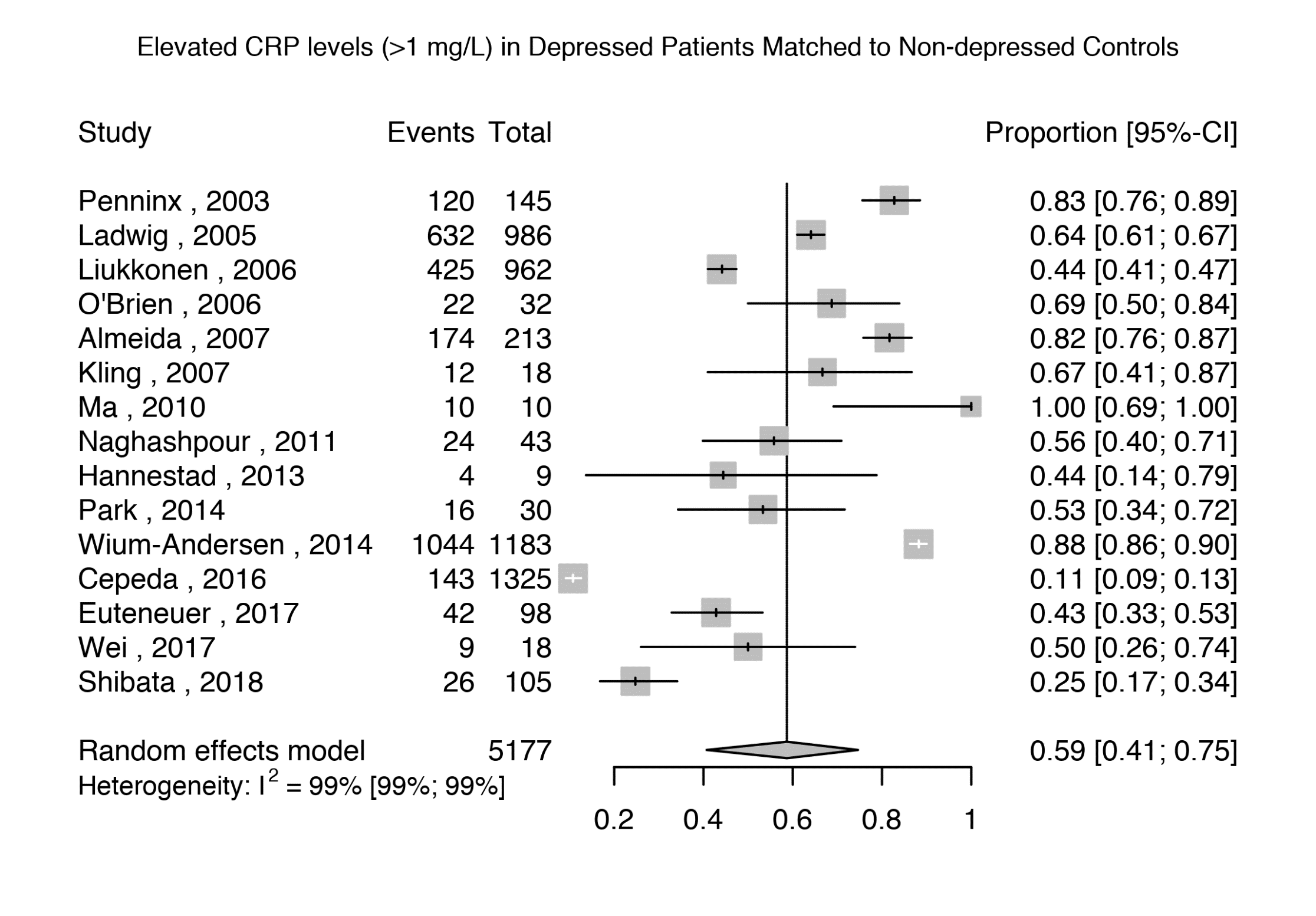


**Supplementary Figure 20: Odds Ratio for Elevated CRP levels (>1 mg/L) in Depressed Patients Compared with Matched Controls, Excluding Poor Quality Studies
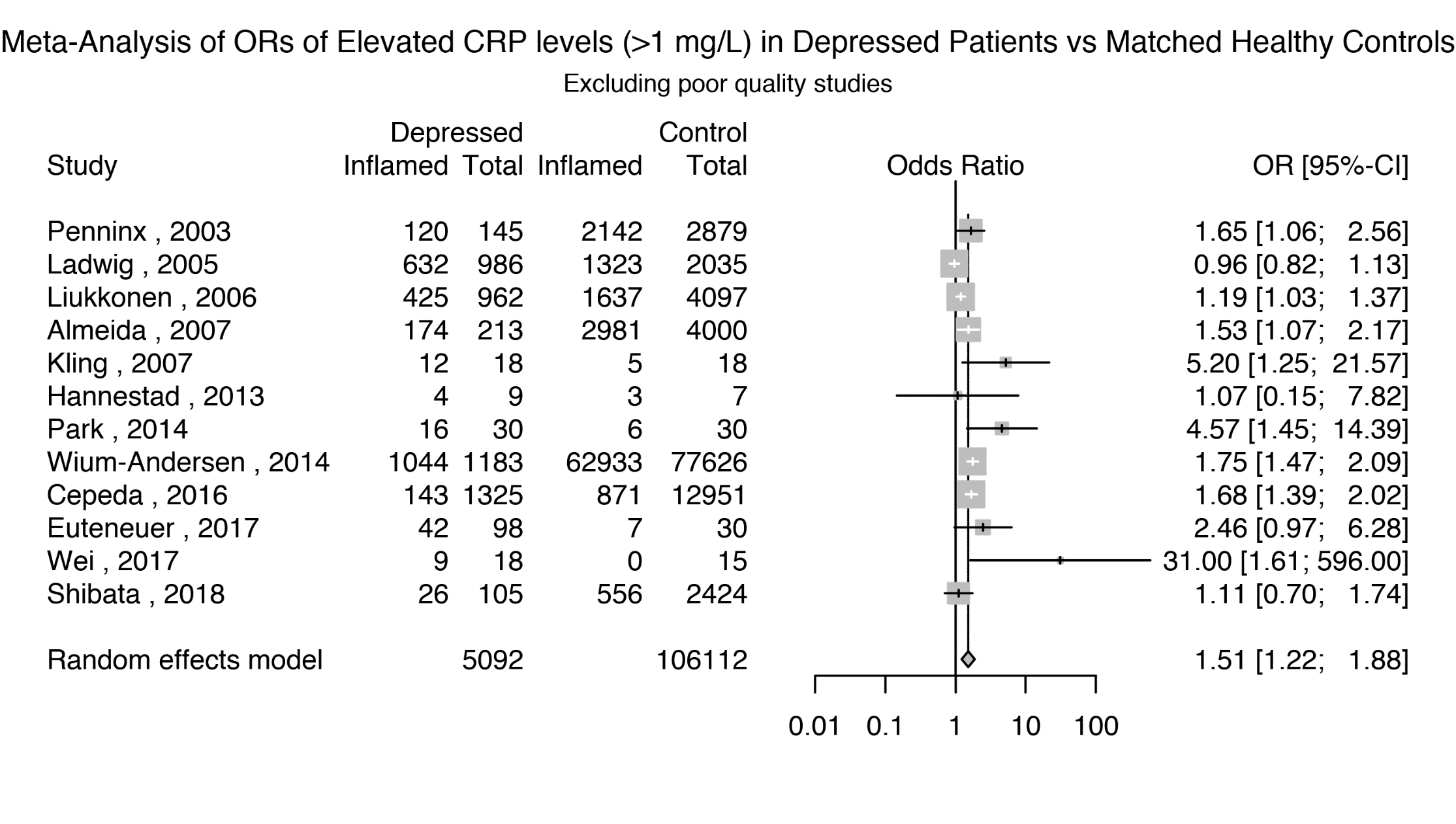
**

**Supplementary Figure 21: Odds Ratio for Elevated CRP levels (>1 mg/L) in Depressed Patients Compared with Matched Controls, Only BMI-Matched Studies
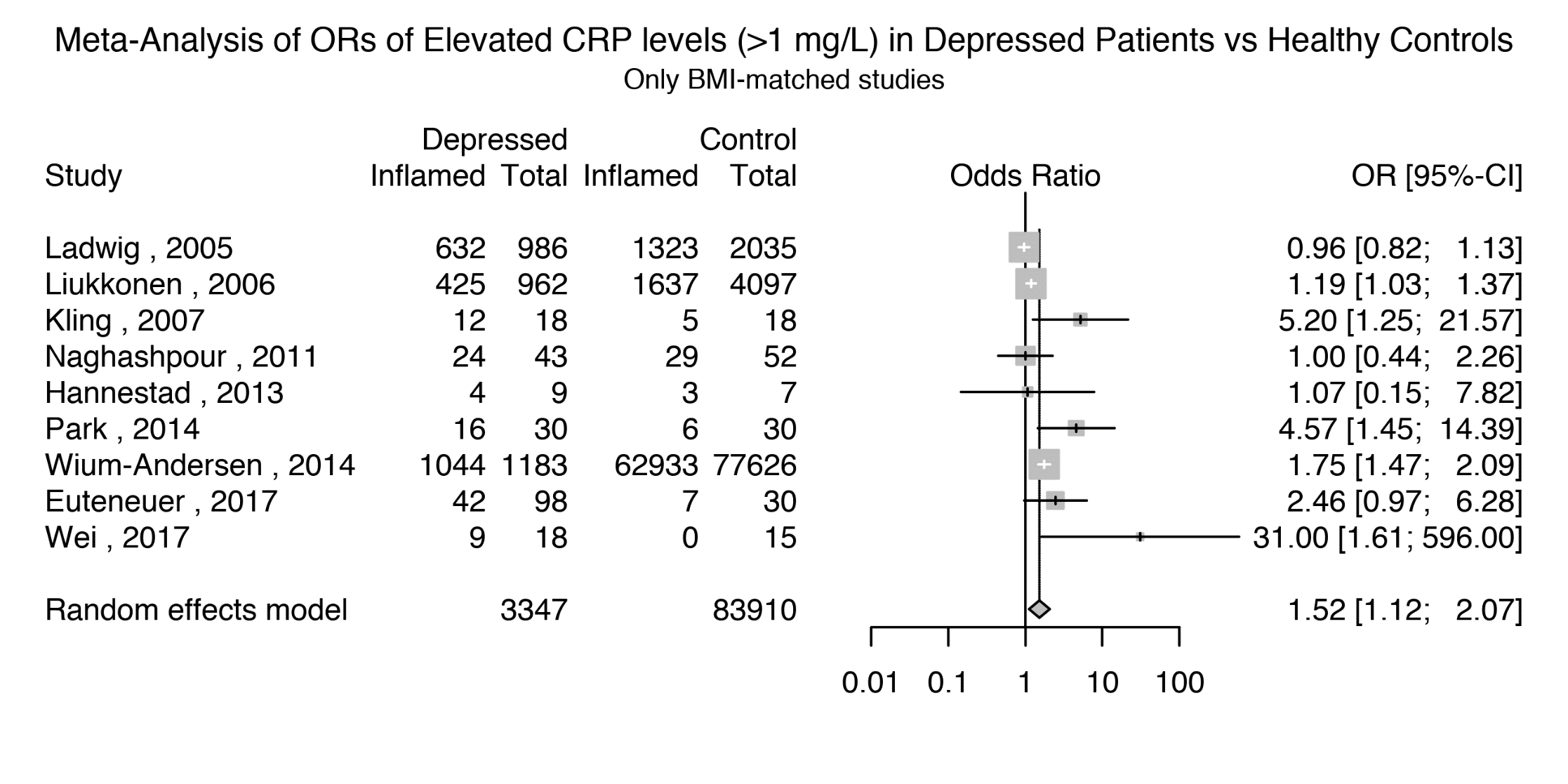
**

**Supplementary Figure 22: Odds Ratio for Elevated CRP levels (>1 mg/L) in Depressed Patients Compared with Matched Controls, Active Depression.
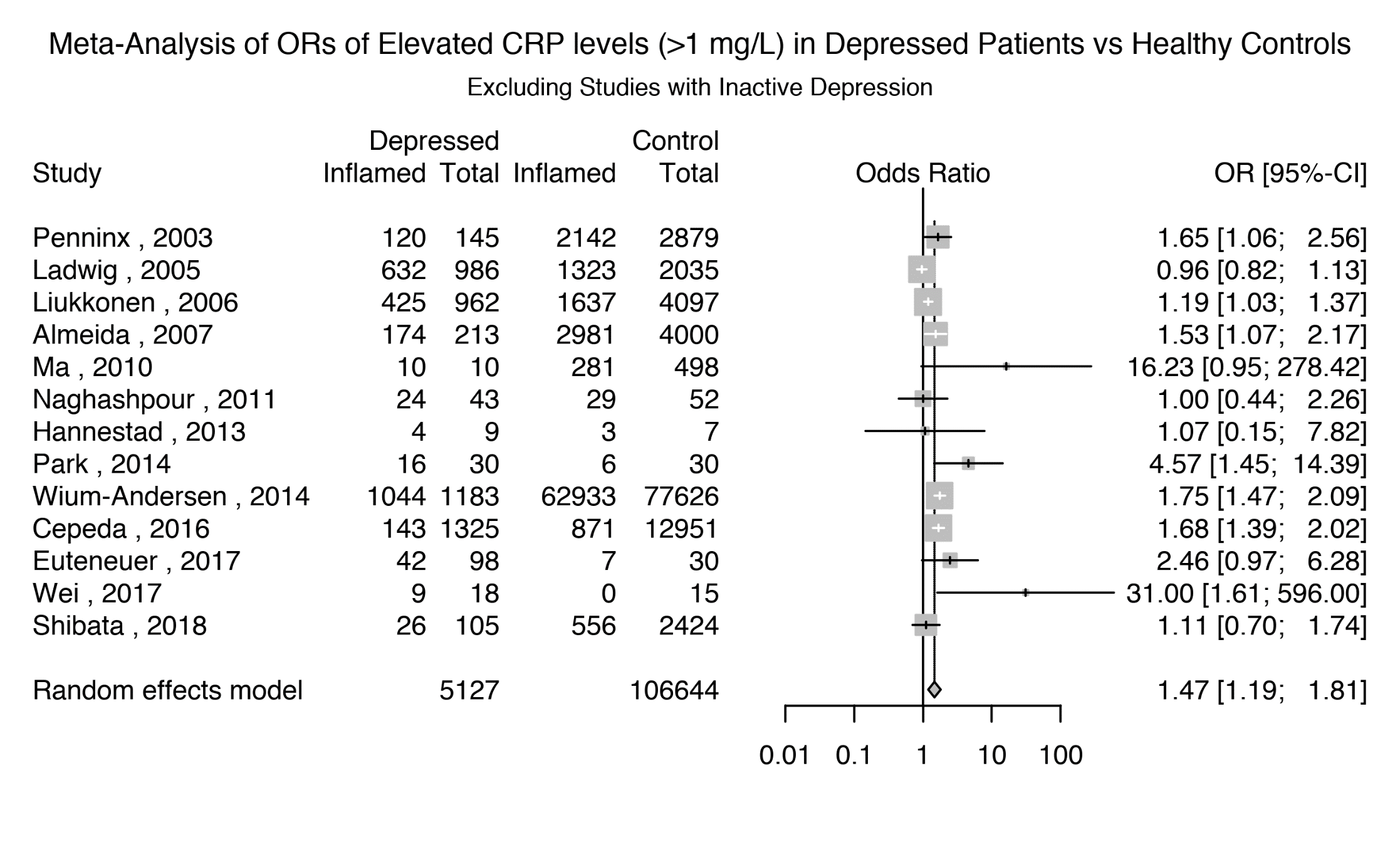
**

**Supplementary Figure 23: Odds Ratio for Elevated CRP levels (>1 mg/L) in Depressed Patients Compared with Matched Controls, studies excluding very high levels of inflammation (>10mg/L)
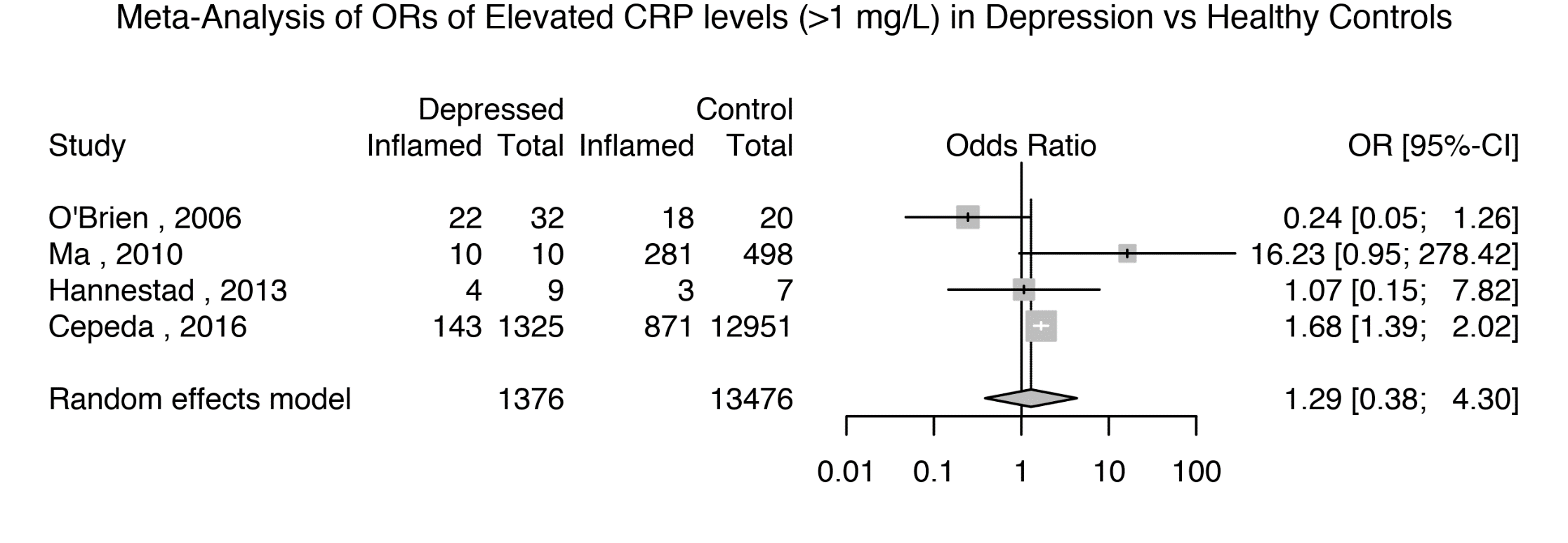
**

**Supplementary Figure 24: Prevalence of Very High CRP (>10mg/L) in Depressed Patients Matched to Healthy Controls**
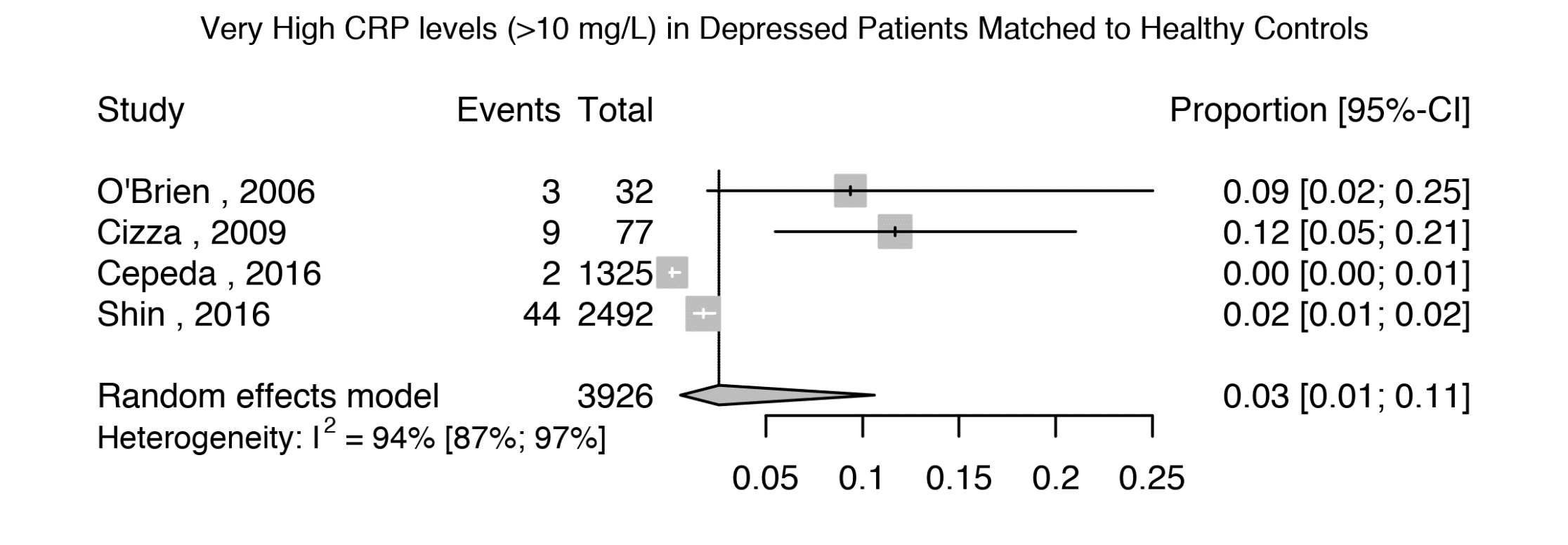


**Supplementary Figure 25: Prevalence of Very High CRP (>10mg/L) in Healthy Controls Matched to Depressed Patients
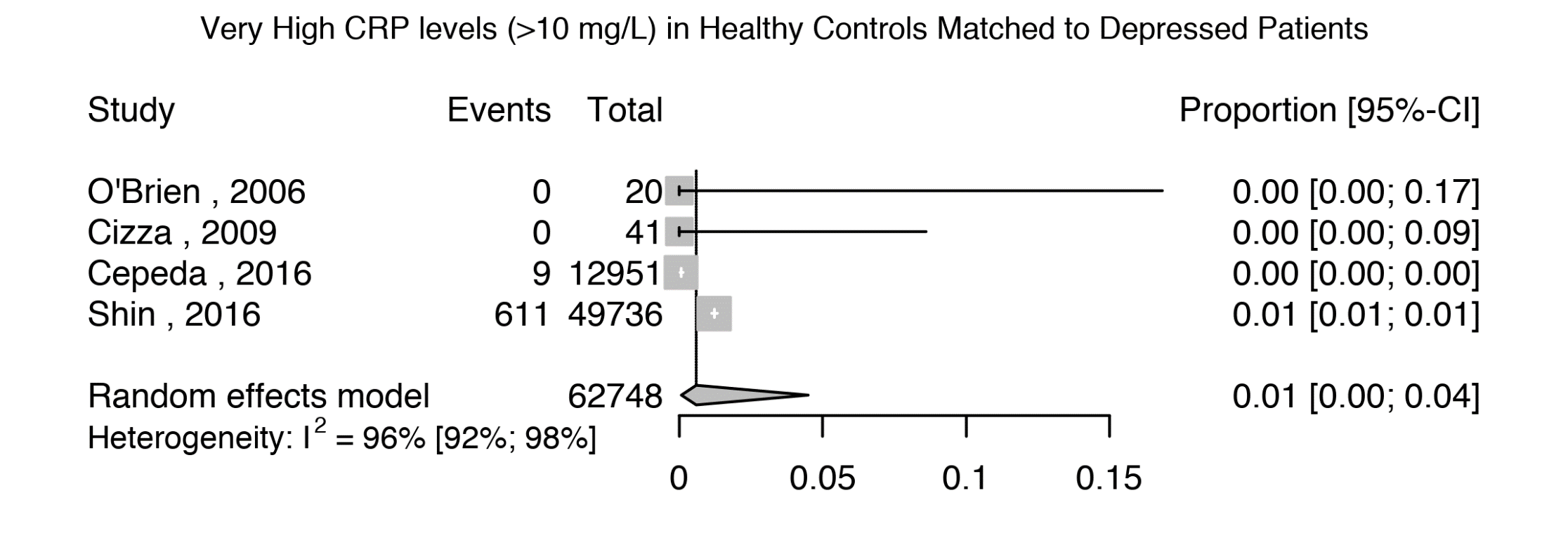
**

**Supplementary Figure 26: Odds Ratio for Very High CRP (>10mg/L) in Depressed Patients Compared with Matched Controls
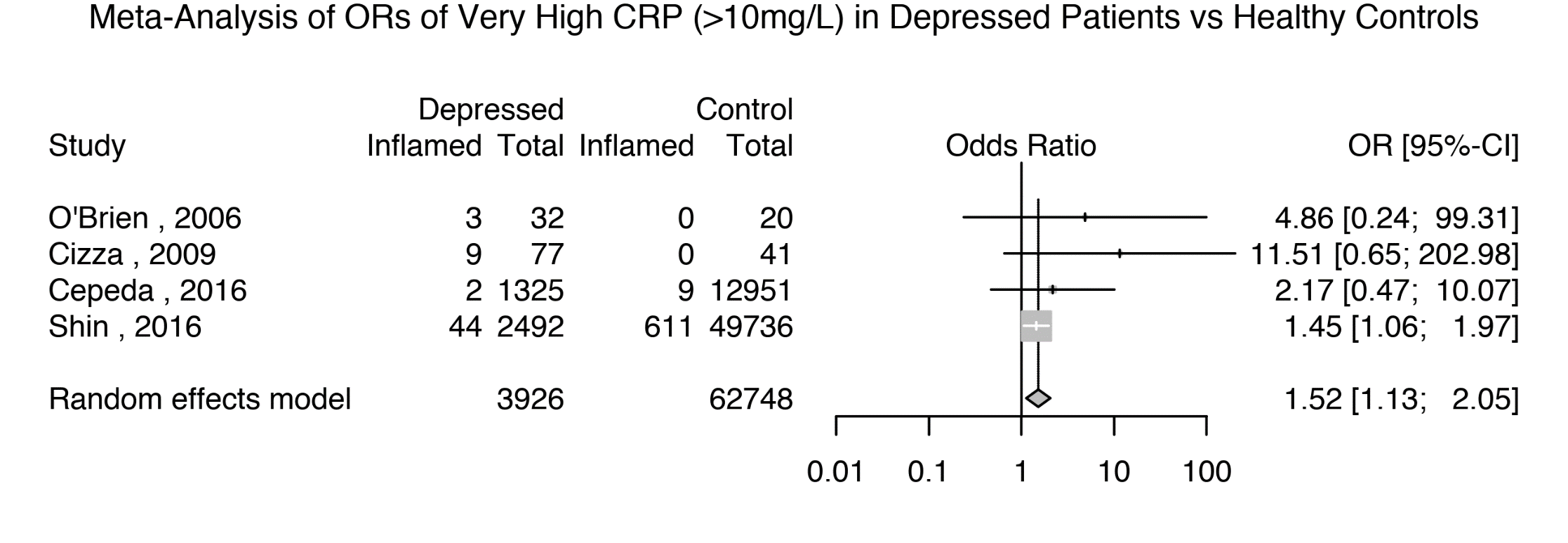
**

# **Supplementary methods**

## **Data Extraction:**

Where data was presented as a plot rather than numerically, we used the WebPlotDigitizer software [http://arohatgi.info/WebPlotDigitizer] to extract study measures. This software has been shown to be a reliable method of data extraction for meta-analyses (Rohatgi, 2016). This was done for 4 studies (Cáceda *et al.*, 2018, Ma *et al.*, 2011, O'brien *et al.*, 2006, Wei *et al.*, 2018).

## **Quality assessment of studies included in meta-analysis.**

The Newcastle Ottawa Scale (Stang, 2010) considers study quality based on three domains: 1) Quality of subject ‘selection’ (cases and controls if available); 2) Quality of ‘comparability’ of cases and controls (defined a priori as matching for age and sex); 3) Quality of ‘exposure’ (defined as use of structured interview to define patients or use of DSM/ICD, and use of structured interview to define absence of mental illness in health controls if available). Item ‘non-response rate’ for Quality of exposure in the scale was not applicable. In order to be able to uniformly assess both prospective and case control studies, as well as studies with no controls, we computed percentage points in each domain (for example, not using control-based scores in studies with no control group).

Thresholds used for converting the NOS rating to Agency for Healthcare Research and Quality - AHRQ - standards (good, fair, and poor):

- Good quality: ≥75% in Selection domain AND ≥50% in Comparability domain AND ≥50% in Outcome domain
- Fair quality: 50% in Selection domain AND ≥50% in Comparability domain AND ≥50% in Outcome domain
- Poor quality: ≤50% in Selection domain OR 0% in Comparability domain OR ≤50% in Outcome domain

# **Supplementary results**

#### **Supplementary Results - Association between Prevalence of Low-grade Inflammation (CRP >3mg/L) and Characteristics of Depressed Patients**

Meta-regression for age was based on 29 studies comprising 11,803 patient (estimate 0.02; z=1.8; p=0.07). Meta-regression for sex was based on 27 studies comprising 11,635 patients (estimate -0.31; z=-0.39; p=0.69). Meta-regression of ethnicity was based on 14 studies comprising 2,266 patients (estimate 0.008; z=1.3; p=0.18). Meta-regression of sample source (inpatients, outpatients, or population-based) was based on 28 studies comprising 11,731 patients (QM=2.54; df=3; p=0.47). Meta-regression of patient BMI was based on 7 studies comprising 1,411 patients (QM= 1.14; df=1; p= 0.29). Only two studies reported prevalence of inflammation in patients with treatment resistant depression, so meta-regression could not be carried out.

#### **Supplementary Results - Prevalence of Elevated CRP levels (>1 mg/L) in Depressed Patients**

Meta-regression for being antidepressant-free at the time of study (including 16 studies; N=5,002) was not significant (estimate -0.007; z=-0.56; p=0.57), suggesting that antidepressant treatment was not correlated with the prevalence of inflammation. Meta-regression for patient age, reported in 23 studies comprising 8,772 patients, was also not significant (estimate 0.03; z=1.9; p=0.05). Meta-regression for patient BMI, reported in 12 studies comprising 3,862 patients, was also not significant (estimate 0.06; z=0.44; p= 0.66). Equally, meta-regression for patient sex, reported in 22 studies comprising 8,687 patients, was not significant (estimate 0.49; z=0.41; p=0.68). The proportion of patients of non-white ethnicity was reported in 13 studies (N=2,007), and was also not a significant meta-regressor (estimate 0.01; k = 13; z=1.4; p=0.16). Finally, a meta-regression on the 24 studies (N=8,855) indicating patient recruitment sources (inpatients, outpatients, longitudinal/population studies) indicated this was not a significant meta-regressor either (QM= 1.98; df=3; p=0.58).

Not enough studies reported the proportion of patients showing high inflammation in treatment resistant patients with depression.

# **Supplementary tables**

## **Supplementary Table 1: Quality Assessment of Included Studies**

| **Study** | **Controls present for CRP >3 mg/L cutoff** | **Setting** | **Selection**  **%** | **Comparability*** | **Exposure**  **%** | **Score**  **%** | **Quality Rating** |
| --- | --- | --- | --- | --- | --- | --- | --- |
| **Legros et al (1985)** | N | inpatient | 100 | 50 | 100 | 83 | Good |
| **Penninx et al (2003)** | Y | prospective | 100 | 50 | 100 | 83 | Good |
| **Ladwig et al (2005)** | Y | population | 100 | 100 | 50 | 83 | Good |
| **Liukkonen et al (2006)** | N | prospective | 100 | 50 | 50 | 67 | Good |
| **O'Brien et al (2006)** | Y | outpatients | 25 | 50 | 50 | 42 | Poor |
| **Almeida et al (2007)** | Y | prospective | 75 | 50 | 50 | 58 | Good |
| **Kling et al (2007)** | Y | outpatients | 75 | 100 | 100 | 92 | Good |
| **Danese et al (2008)** | Y | prospective | 100 | 50 | 100 | 83 | Good |
| **Nilsson et al (2008)** | N | outpatients | 100 | 50 | 100 | 83 | Good |
| **Cizza et al (2009)** | N | outpatients | 100 | 100 | 100 | 100 | Good |
| **Harley et al (2010)** | N | outpatients | 100 | 0 | 100 | 67 | Poor |
| **Ma et al (2010)** | Y | outpatients | 50 | 0 | 100 | 50 | Poor |
| **Naghashpour et al (2011)** | Y | outpatients | 25 | 100 | 100 | 75 | Poor |
| **Hannestad et al (2013)** | Y | outpatients | 50 | 100 | 100 | 83 | Fair |
| **Raison et al (2013)** | N | outpatients | 100 | 100 | 100 | 100 | Good |
| **Shanahan et al (2013)** | N | prospective | 100 | 50 | 100 | 83 | Good |
| **Park et al (2014)** | Y | outpatients | 100 | 100 | 100 | 100 | Good |
| **Uher et al (2014)** | N | outpatients | 100 | 100 | 100 | 100 | Good |
| **Wium-Andersen et al (2014)** | Y | prospective | 75 | 100 | 100 | 58 | Good |
| **Wysokinski et al (2014)** | N | inpatient | 0 | 100 | 100 | 67 | Poor |
| **Courtet et al (2015)** | N | inpatient | 100 | 100 | 100 | 100 | Good |
| **Cepeda et al (2016)** | Y | population | 75 | 50 | 100 | 75 | Good |
| **Haroon et al (2016)** | N | outpatients | 50 | 100 | 100 | 83 | Fair |
| **Rapaport et al (2016)** | N | outpatients | 50 | 100 | 100 | 83 | Fair |
| **Shin et al (2016)** | Y | outpatients | 100 | 0 | 100 | 67 | Poor |
| **Ekinci et al (2017)** | Y | inpatient | 25 | 100 | 50 | 58 | Poor |
| **Euteneuer et al (2017)** | N | outpatients | 75 | 100 | 100 | 92 | Good |
| **Gallagher et al (2017)** | Y | prospective | 75 | 100 | 100 | 58 | Good |
| **Horsdal et al (2017)** | N | population | 75 | 100 | 100 | 92 | Good |
| **Jha et al (2017)** | N | outpatients | 100 | 100 | 100 | 100 | Good |
| **Wei et al (2017)** | Y | inpatient | 75 | 100 | 50 | 75 | Good |
| **Caceda et al (2018)** | N | inpatient | 100 | 100 | 100 | 100 | Good |
| **Chamberlain et al (2018)** | Y | outpatients | 100 | 100 | 100 | 100 | Good |
| **Felger et al (2018)** | N | outpatients | 100 | 100 | 100 | 100 | Good |
| **Osimo et al (2018)** | N | inpatient | 100 | 100 | 100 | 100 | Good |
| **Porcu et al (2018)** | N | outpatients | 100 | 50 | 100 | 83 | Good |
| **Shibata et al (2018)** | N | population | 100 | 100 | 100 | 67 | Good |

***Comparability** scored for age and sex reporting/matching

Thresholds used for converting the NOS rating to Agency for Healthcare Research and Quality - AHRQ - standards (good, fair, and poor):

- Good quality: ≥75% in Selection domain AND ≥50% in Comparability domain AND ≥50% in Outcome domain
- Fair quality: 50% in Selection domain AND ≥50% in Comparability domain AND ≥50% in Outcome domain
- Poor quality: ≤50% in Selection domain OR 0% in Comparability domain OR ≤50% in Outcome domain

## **Supplementary Table 2: Matching criteria for case-controls studies defining inflammation as CRP >3mg/L**

| Author | Year | N depressed | N controls | Matching variable | | | | |
| --- | --- | --- | --- | --- | --- | --- | --- | --- |
|  |  |  |  | Age | Sex | BMI | Smoking | Ethnicity |
| Penninx | 2003 | 145 | 2879 | ✓ | ✗ | NA | ✓ | ✓ |
| Ladwig | 2005 | 986 | 2035 | ✓ | ✓ | ✓ | ✓ | NA |
| O'Brien | 2006 | 32 | 20 | ✓ | ✗ | NA | NA | NA |
| Almeida | 2007 | 213 | 4000 | ✗ | ✓ | ✗ | NA | NA |
| Kling | 2007 | 18 | 18 | ✓ | ✓ | ✓ | ✓ | ✓ |
| Danese | 2008 | 109 | 673 | ✓ | ✗ | NA | ✗ | ✓ |
| Ma | 2010 | 10 | 498 | NA | ✗ | NA | NA | NA |
| Naghashpour | 2011 | 43 | 52 | ✓ | ✓ | ✓ | NA | ✓ |
| Hannestad | 2013 | 9 | 7 | ✓ | ✓ | ✓ | ✓ | ✓ |
| Park | 2014 | 30 | 30 | ✓ | ✓ | ✓ | ✓ | NA |
| Wium-Andersen | 2014 | 1183 | 77626 | ✗ | ✗ | ✓ | ✗ | ✓ |
| Cepeda | 2016 | 1325 | 12951 | ✓ | ✗ | ✗ | ✗ | NA |
| Shin | 2016 | 2492 | 49736 | ✗ | ✗ | ✗ | ✗ | NA |
| Ekinci | 2017 | 139 | 50 | ✓ | ✓ | ✓ | ✓ | ✓ |
| Gallagher | 2017 | 811 | 5084 | ✓ | ✓ | ✗ | NA | NA |
| Wei | 2017 | 18 | 15 | ✓ | ✓ | ✓ | ✗ | ✓ |
| Chamberlain | 2018 | 198 | 54 | ✓ | ✓ | ✓ | ✓ | NA |

# **Supplementary references**

**Cáceda, R., Griffin, W. S. T. & Delgado, P. L.** (2018). A probe in the connection between inflammation, cognition and suicide. *Journal of psychopharmacology* **32**, 482-488.

**Ma, Y., Chiriboga, D. E., Pagoto, S. L., Rosal, M. C., Li, W., Merriam, P. A., Hébert, J. R., Whited, M. C. & Ockene, I. S.** (2011). Association between depression and C-reactive protein. *Cardiology research and practice* **2011**.

**O'brien, S. M., Scott, L. V. & Dinan, T. G.** (2006). Antidepressant therapy and C-reactive protein levels. *The British Journal of Psychiatry* **188**, 449-452.

**Rohatgi, A.** (2016). WebPlotDigitizer. p. <http://arohatgi.info/WebPlotDigitizer>.

**Stang, A.** (2010). Critical evaluation of the Newcastle-Ottawa scale for the assessment of the quality of nonrandomized studies in meta-analyses. *Eur J Epidemiol* **25**, 603-5.

**Wei, L., Du, Y., Wu, W., Fu, X. & Xia, Q.** (2018). Elevation of plasma neutrophil gelatinase-associated lipocalin (NGAL) levels in schizophrenia patients. *Journal of affective disorders* **226**, 307-312.
